# Primary Immune Regulatory Disorders With an Autoimmune Lymphoproliferative Syndrome-Like Phenotype: Immunologic Evaluation, Early Diagnosis and Management

**DOI:** 10.3389/fimmu.2021.671755

**Published:** 2021-08-10

**Authors:** Marta López-Nevado, Luis I. González-Granado, Raquel Ruiz-García, Daniel Pleguezuelo, Oscar Cabrera-Marante, Nerea Salmón, Pilar Blanco-Lobo, Nerea Domínguez-Pinilla, Rebeca Rodríguez-Pena, Elena Sebastián, Jaime Cruz-Rojo, Peter Olbrich, Jesús Ruiz-Contreras, Estela Paz-Artal, Olaf Neth, Luis M. Allende

**Affiliations:** ^1^Immunology Department, University Hospital 12 de Octubre, Madrid, Spain; ^2^Research Institute Hospital 12 Octubre (imas12), Madrid, Spain; ^3^Immunodeficiency Unit, Department of Pediatrics, University Hospital 12 de Octubre, Madrid, Spain; ^4^Immunology Department, Centre Diagnòstic Biomèdic, Hospital Clínic, Barcelona, Spain; ^5^Paediatric Infectious Diseases, Rheumatology and Immunology Unit, University Hospital Virgen del Rocío, Institute of Biomedicine, Biomedicine Institute (IBiS)/University of Seville/Superior Council of Scientific Investigations (CSIC), Seville, Spain; ^6^Pediatric Hematology and Oncology Unit, Toledo Hospital Complex, Toledo, Spain and University Hospital 12 de Octubre, Madrid, Spain; ^7^Immunology Department, University Hospital La Paz, Madrid, Spain; ^8^Hematology and Hemotherapy Unit, University Children’s Hospital Niño Jesús, Madrid, Spain; ^9^Endocrine Unit, Department of Pediatrics, University Hospital 12 de Octubre, Madrid, Spain; ^10^School of Medicine, Complutense University of Madrid, Madrid, Spain

**Keywords:** ALPS, ALPS-like, autoimmunity, lymphoproliferation, malignancy, immune dysregulation

## Abstract

Primary immune regulatory disorders (PIRD) are associated with autoimmunity, autoinflammation and/or dysregulation of lymphocyte homeostasis. Autoimmune lymphoproliferative syndrome (ALPS) is a PIRD due to an apoptotic defect in Fas-FasL pathway and characterized by benign and chronic lymphoproliferation, autoimmunity and increased risk of lymphoma. Clinical manifestations and typical laboratory biomarkers of ALPS have also been found in patients with a gene defect out of the Fas-FasL pathway (ALPS-like disorders). Following the Preferred Reporting Items for Systematic Reviews and Meta-analyses (PRISMA), we identified more than 600 patients suffering from 24 distinct genetic defects described in the literature with an autoimmune lymphoproliferative phenotype (ALPS-like syndromes) corresponding to phenocopies of primary immunodeficiency (PID) (*NRAS, KRAS*), susceptibility to EBV (*MAGT1, PRKCD, XIAP, SH2D1A, RASGRP1, TNFRSF9*), antibody deficiency (*PIK3CD* gain of function (GOF)*, PIK3R1* loss of function (LOF)*, CARD11* GOF), regulatory T-cells defects (*CTLA4, LRBA, STAT3* GOF*, IL2RA, IL2RB, DEF6*), combined immunodeficiencies (*ITK, STK4*), defects in intrinsic and innate immunity and predisposition to infection (*STAT1* GOF, *IL12RB1*) and autoimmunity/autoinflammation (*ADA2, TNFAIP3,TPP2, TET2*). CTLA4 and LRBA patients correspond around to 50% of total ALPS-like cases. However, only 100% of CTLA4, PRKCD, TET2 and NRAS/KRAS reported patients had an ALPS-like presentation, while the autoimmunity and lymphoproliferation combination resulted rare in other genetic defects. Recurrent infections, skin lesions, enteropathy and malignancy are the most common clinical manifestations. Some approaches available for the immunological study and identification of ALPS-like patients through flow cytometry and ALPS biomarkers are provided in this work. Protein expression assays for NKG2D, XIAP, SAP, CTLA4 and LRBA deficiencies and functional studies of AKT, STAT1 and STAT3 phosphorylation, are showed as useful tests. Patients suspected to suffer from one of these disorders require rapid and correct diagnosis allowing initiation of tailored specific therapeutic strategies and monitoring thereby improving the prognosis and their quality of life.

## Introduction

Human Inborn Errors of Immunity (IEI), also referred to as Primary Immunodeficiencies (PID) are a group of heterogeneous genetic diseases that disrupt the function of the immune system. Patients manifest increased susceptibility to develop a broad or narrow constellation of infectious, autoimmune, autoinflammatory, allergic, and/or malignant phenotypes. IEI represent more than 400 distinct disorders with more than 430 different gene defects listed in the classical and phenotypical 2019 International Union of Immunological Societies (IUIS) classification (1, 2). These diseases are mainly monogenic, but increasingly more complex genetic cases such as incomplete penetrance, polygenicity, transcriptional, somatic and epigenetic alterations are being described (3–6).

According to the European Society for Immunodeficiencies (ESID), Primary Immune Regulatory Disorders (PIRD) represent approximately 5.3% of IEI and currently comprise 45 disease-causing genes divided into four categories: hemophagocytic lymphohistiocytosis (HLH), susceptibility to EBV, syndromes with autoimmunity and immune dysregulation with colitis (1, 2). Autoimmune lymphoproliferative syndrome (ALPS), a well-known syndrome with autoimmunity, is a PIRD characterized by chronic and benign lymphoproliferation, autoimmune manifestations and an increased risk of lymphoma due to a defect in lymphocyte apoptosis (7, 8). Expanded CD3+TcRαβ+CD4-CD8- double negative T-cells (DNT) are a hallmark of ALPS patients who can also present elevated plasma biomarkers including interleukin-10 (IL-10), soluble FasL (sFasL) (diminished in patients with defect in *FASL*) and vitamin B12 (9). Genetic causes comprise the apoptotic Fas-FasL pathway components (*FAS, FASL, FADD, CASP10 and CASP8*), being germinal and somatic mutations in *FAS* gene (ALPS-FAS and ALPS-sFAS, respectively) the most common cause of ALPS (60% and 15%, respectively) (7, 8, 10–14). However, approximately 20% of patients with ALPS do not have an identifiable genetic mutation (ALPS-U) (9).

Advances in NGS technologies have enabled an expanding list of monogenic defects in PIRD. Numerous genetic defects outside the Fas-FasL pathway mimicking an ALPS phenotype have been identified (ALPS-like syndromes).

Clinically, autoimmunity and lymphoproliferation is a common finding also in patients with common variable immunodeficiency (CVID) without known genetic defect and therefore cannot be used as a diagnostic marker. Therefore, ALPS immunophenotyping, biomarkers and genetic testing by NGS could be useful for the identification of patients with an overlapping clinical phenotype such as ALPS, ALPS-like, CVID, combined immunodeficiency (CID) and immunodysregulation, polyendocrinopathy, enteropathy, X-linked syndrome (IPEX) disorders (15–17). To date, thirteen genes have been related with ALPS-like syndrome in the literature: phenocopies of PID (*NRAS* and *KRAS*), susceptibility to EBV (*MAGT1, PRKCD*), regulatory T-cells defects (*CTLA4, LRBA, STAT3* GOF), combined immunodeficiencies (*ITK, STK4*), defects in intrinsic and innate immunity and predisposition to infection (*STAT1* GOF, *IL12RB1*) and autoimmunity/autoinflammation (*ADA2, TET2*) (18–29).

The approach to the diagnosis and initial management of these immune dysregulation disorders is often similar. However, identification of specific genetic defects and validation by immune evaluation can facilitate the use of specific targeted treatments for the defects that share similar pathophysiology mechanism (30–33).

In the manuscript, we show clinical and immunological characteristics of ALPS-like patients with the aim to identify specific and overlapping phenotypes in the amazing setting of numerous novel disorders, in which a basic immune evaluation can be misleading. Since the broader application of NGS has provided a greater knowledge within this field, rendering the diagnostic approach and the management of these disorders challenging, this study aims to provide important information to identify specificities of each group of disorders and it will hopefully present an important and practical tool to orientate the first steps of the diagnostic process.

## Methods

### Case Reports Search Strategy and Study Selection

The literature search was conducted in accordance with the Preferred Reporting Items for Systematic Reviews and Meta-analyses (PRISMA) guidelines (34). Clinical ALPS-like criteria were immune dysregulation defined as autoimmunity and lymphoproliferation (17, 29, 35). The genes in which genetic defects gave rise to an ALPS-like phenotype, excluding those already described in the Fas-FasL pathway, were selected based on the IUIS classification (1, 2) and a literature search in MEDLINE (pubmed), Google scholar databases, Web Of Science (WOS) and congress communications. The following research keywords in English were used: (“ALPS”), (“ALPS-like”), (“Autoimmune lymphoproliferative syndrome”), (“autoimmune lymphoproliferative syndrome-like”), (“autoimmunity” AND “lymphoproliferation”), (“immune dysregulation” AND “autoimmunity”). Once ALPS-like-causing genes were identified, we classified them into the 10 groups of IUIS classification (1, 2). All types of publications reporting patients, in English or in Spanish, published until October 26, 2020, were collected and total of reported patients were counted (**Supplementary Tables 1**, **2**). Those patients described in more than one article or cohort were only counted in the first report. Total cases were filtered according to the clinical criteria for ALPS-like. Those patients who presented lymphoproliferation in the form of lymphadenopathy, splenomegaly, or hepatomegaly together with autoimmune features were selected and clinical, laboratory, and molecular data were collected in a database.

### Laboratory Studies

We show diverse functional studies by flow cytometry of not reported ALPS-like patients, diagnosed in the authors’ laboratories. Immunophenotyping of T, B and NK compartments, protein expression (NKG2D, XIAP, SAP, CTLA4 and LRBA) and phosphorylation assays (AKT, STAT3 and STAT1) could be rapid and useful to direct the diagnosis in some patients. Serial monitoring of serum levels of soluble CD25 (sCD25) (R&D system, Abingdon, UK) could be very useful as a measure of disease activity and response to therapy. For further details, see methods in **Supplementary Material** and **Supplementary Table 3**.

## Results

### ALPS-Like Related Genes

**Figure 1:**
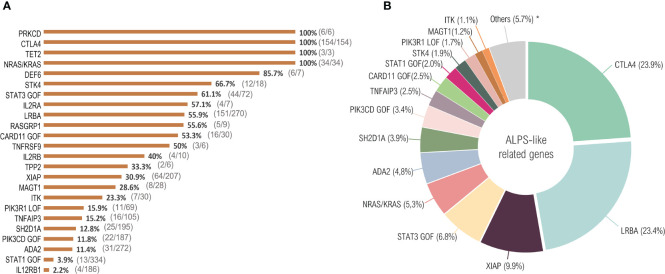
ALPS-like related cases. Twenty-four distinct genetic defects with immune dysregulation, lymphoproliferation and autoimmunity were identified in the literature until October 2020. More than 2000 total cases were reported. After filtering according to the ALPS-like inclusion criteria, 645 patients were selected. **(A)** percentage of patients that fulfill clinical ALPS-like phenotype criteria. The number of ALPS-like patients with respect to the total number of patients counted in each genetic defect is indicated in parentheses. **(B)** ALPS-like related genes prevalence. *ALPS-like related genes with a prevalence less than 1%: *PRKCD* (0.9%), *DEF6* (0.9%), *RASGRP1* (0.8%), *IL2RA* (0.6%), *IL2RB* (0.6%), *TNFRSF9* (0.5%), *TET2* (0.5%), *TPP2* (0.3%).

The search identified 526 publications describing 2245 unique patients suffering from defects in 24 distinct genes, distributed between the 10 large groups of the IUIS classification: phenocopies of PID (*NRAS, KRAS*), susceptibility to EBV (*MAGT1, PRKCD, XIAP, SH2D1A, RASGRP1, TNFRSF9*), antibody deficiency (*PIK3CD* GOF*, PIK3R1* LOF*, CARD11* GOF), regulatory T-cells defects (*CTLA4, LRBA, STAT3* GOF*, IL2RA, IL2RB, DEF6*), combined immunodeficiencies (*ITK, STK4*), defects in intrinsic and innate immunity and predisposition to infection (*STAT1* GOF, *IL12RB1*) and autoimmunity/autoinflammation (*ADA2, TNFAIP3, TPP2, TET2*). **Supplementary Figure 1** summarizes the PRISMA diagram. Of total counted, 645 patients were filtered fulfilling the clinical ALPS-like inclusion criteria (**Figure 1** and **Supplementary Table 1**).

All PRKCD, CTLA4, TET2 and NRAS/KRAS patients presented clinically with lymphoproliferation and autoimmunity, considering all those reported cases (n=197) as ALPS-like patients (**Figure 1A**). ALPS-like phenotype was seen in a significant percentage of patients suffering, among others, from mutations in *STK4, STAT3 *(GOF)*, LRBA or CARD11* (GOF) (66.7%, 61.1%, 55.9% and 53.3%, respectively, corresponding to 223 out of 390 patients), while the combination of autoimmunity and lymphoproliferation is quite rare in others. Very few cases of STAT1 GOF or IL12RB1 deficiency were suspected of ALPS-like (3.9% and 2.2%, respectively, corresponding to 17 out of 520 patients) (**Figure 1A**).

CTLA4, together with LRBA, is the most common cause of ALPS-like. Both disorders represent around the 50% of total filtered cases, while none of the rest of disorders exceed the 10% of total representation (**Figure 1B**).

To reduce the ALPS-like subgroups and due to similar immunopathology pathways, the patients have been grouped into Epstein Barr Virus (EBV) susceptibility, regulatory T-cells defect and others. Clinical and laboratory features as well as treatment are summarized in **Table 1**.

**Table 1:** Clinical and laboratory features of ALPS-like patients.

	EBV susceptibility							
	PRKCD	MAGT1	XIAP	SH2D1A	RASGRP1	TNFRSF9	PIK3CD	PIK3R1
**IUIS classification**	Immune dysregulation	Immune dysregulation	Immune dysregulation	Immune dysregulation	Immune dysregulation	Immune dysregulation	Antibody deficiencies	Antibody deficiencies
**Inheritance**	AD LOF	XL	XL	XL	AR	AR	AD GOF	AD GOF
**Clinical features**								
Lymphoproliferation	S, H, L	S, H, L	S, H, L	S, H, L	S, H, L	S, H, L	S, H, L	S, H, L
Autoimmunity	Cytopenia, SLE, APS, vasculitis	Cytopenia	Cytopenia, AIE	Cytopenia	Cytopenia	Cytopenia	Cytopenia, SLE	Cytopenia, AIT, vasculitis
Malignancy	NR	HL and NHL	NR	NHL	HL	HL	HL	HL, NHL
Associated features	RI, skin lesions, diarrhea	RI, neurological affection	RI, HLH, IBD, skin lesions, JIA, GLILD	RI, HLH, neurological affection, skin lesions, diarrhea	RI, skin lesions, diarrhea, HLH	RI, HLH	RI, skin lesions, diarrhea, HLH	RI, skin lesions, diarrhea
**ALPS parameters**								
DNT	Normal	Normal	NR	NR	Normal	NR	Normal/moderate high	NR
Vitamin B12	NR	Normal	NR	NR	NR	NR	NR	NR
sFASL	NR	Normal	NR	NR	NR	NR	NR	NR
IL-10	NR	Normal	NR	NR	NR	NR	NR	NR
IgG level	Low/Normal/High	Low/Normal	Low / normal	Low	Low/Normal/High	Low	Low/Normal	Low
IgA level	Normal	Low/Normal	Low / normal	Low	Normal/High	Low	Low/Normal	Low
IgM level	Normal/High	Low/Normal	Low / normal	Normal/High	Normal/High	Normal	High	Normal/High
**Immunophenotype**								
T-cell compartment	Normal	CD4 lymphopenia. Inverted CD4:CD8 ratio.	Inverted CD4:CD8 ratio. High T memory	Inverted CD4:CD8 ratio.	CD4 lymphopenia. Inverted CD4:CD8 ratio	Low TFH. Low Treg	CD4 lymphopenia. Inverted CD4:CD8 ratio. Low naïve, senescent CD8	Inverted CD4:CD8 ratio. Low naïve, senescent CD8 (**Figure 4F**)
B-cell compartment	B lymphocytosis. High CD21low and BT. Low BM	B lymphopenia. Low BSM, normal/high BT	B lymphopenia	Low BM	Normal	Low BM, high BT	Low BM, high BT	Low BM, high BT
NK-cell compartment	Low/normal NK	Low NK	Low/ normal NK	Low NK	Normal	Low NK	Normal	Normal
Other	NR	NR	Low iNKT	Low iNKT	NR	NR	NR	NR
**Clinical functional assays**	High B proliferation. Low NK activity. Decreased/ normal T proliferation. Poor vaccine response	Low/normal NK degranulation. Low NKG2D expression (**Figure 4D**). Poor vaccine response	Low XIAP expression (**Figure 4C**)	Low SAP expression (**Figure 4B**). Low NK activity. Poor vaccine response	Impaired T proliferation	Impaired T and B proliferation. Poor vaccine response	Impaired/ normal T proliferation. Poor vaccine response	Impaired/ normal T proliferation. Poor vaccine response
**Research functional assays**	–	–	Impaired NOD2 signaling in monocytes	–	Impaired ERK activation	–	High AKT, S6 phosphorylation. Upregulated *IGF1, TP53, HIF1A* expression	High AKT, S6 and FOXO1/3A phosphorylation (**Figure 4E**)
**Treatment**	Hydroxychloroquine. TMP/SMX. IVIG. Steroids. Rapamycin. Rituximab. MMF	IVIG. Rituximab. Oral Mg^2+^.Ganciclovir.	Steroids, immunosuppressive drugs. Adalimumab/ infliximab. HSCT	Steroids, immunosuppressive drugs. IVIG. HSCT	Steroids. IVIG. HSCT	IVIG. Rituximab. HSCT	Steroids. mTOR inhibitors. IVIG. Leniolisib. HSCT	Steroids. mTOR inhibitors. IVIG. Leniolisib. HSCT

	EBV susceptibility				Tregs defects			
	ITK		STK4		STAT3	CTLA4	LRBA	CD25
**IUIS classification**	Combined immunodeficiency		Combined immunodeficiency		Immune dysregulation	Immune dysregulation	Immune dysregulation	Immune dysregulation
**Inheritance**	AR		AR		AD GOF	AD	AR	AR
**Clinical features**								
Lymphoproliferation	S, H, L		S, H, L		S, H, L	S, H, L	S, H, L	S, H, L
Autoimmunity	Cytopenia, vitiligo		Cytopenia, polyarthritis		Cytopenia, AIT, AIE, CD	Cytopenia, AIE, AIEC	Cytopenia, AIE	Cytopenia
Malignancy	HL		HL, NHL		HL*	HL, NHL, gastric cancer	HL, NHL	NR
Associated features	RI, skin lesions, ILD, hearing defect, HLH		RI, CMC, skin lesions, hearing defect, nephropathy, cardiomyopathy		ILD, IBD, skin lesions, liver disease, arthritis	RI, IBD, skin lesions, GLILD, Lymphocytic infiltration of nonlymphoid organs, neurological affection, arthritis, HLH	RI, IBD, skin lesions, GLILD, Lymphocytic infiltration of nonlymphoid organs, neurological affection, arthritis, HLH	RI, skin lesions, diarrhea, polyendocrinopathy
**ALPS parameters**								
DNT	High		High		Normal/High	Normal/High	Normal/High	Normal/High
Vitamin B12	High		NR		Normal/moderate high	Normal	Normal	NR
sFASL	NR		NR		High	Normal	Normal/High	NR
IL-10	NR		NR		NR	NR	Normal	NR
IgG level	Low/Normal		Low/Normal/ High		Low/Normal	Low/Normal	Low/Normal	Normal
IgA level	Low/Normal		Normal/ High		Low/Normal/High	Low/Normal	Low/Normal	Normal/High
IgM level	Low/Normal/High		Low/Normal/High		Low/Normal/High	Low/Normal	Low/Normal	Normal
**Immunophenotype**								
T-cell compartment	CD4 lymphopenia. Low naïve. Low CD8 CM and TEMRA		CD3 lymphopenia. Low T naïve.		CD3 lymphopenia. Low CD4 naïve. High CD4 memory. Low Th17. Low/normal Tregs	CD3 lymphopenia. Low CD4 naïve. Low/normal Tregs. High cTFH	CD3 lymphopenia. Low CD4 naïve. Low/normal Tregs (**Figure 5B**). High cTFH (**Figure 5E**)	Inverted CD4:CD8 ratio. Normal Tregs values. Low T naïve, high T memory
B-cell compartment	Normal		B lymphopenia, Low BM and BSM, high BT		High B naïve, low BSM, high CD21low	B lymphopenia. High B naïve, low BSM, high CD21low	Low BSM, high BT	High B naïve, low BT and BM
NK-cell compartment	Normal		Low NK		Low NK	Low/normal NK	Low/normal NK	Low NK
Other	Low iNKT		NR		Low pDC	NR	NR	NR
**Clinical functional assays**	Impaired T proliferation.		Low T proliferation, high apoptosis. Poor vaccine response		High sCD25 (**Figure 7**). Poor vaccine response	Impaired Treg suppression, low CTLA4 expression (**Figure 5C**). Poor vaccine response	Impaired Treg suppression, low LRBA expression (**Figure 5D**). High sCD25 (**Figure 7**). Poor vaccine response	Low CD25 expression/sCD25. Rescue of T proliferation with IL-2. Poor vaccine response
**Research functional assays**	Low S6 phosphorylation		Low STK4 and FOXO3a expression.		Normal/high STAT3 and low STAT5 and STAT1 phosphorylation (**Figure 5F**). High *SOCS3* expression	CTLA4 transendocytosis defect	–	Low STAT5 phosphorylation.
**Treatment**	Steroids. Rituximab. Foscarnet/ ganciclovir.		Steroids, immunosuppressive drugs. IVIG. Rituximab. HSCT		Steroids. IVIG. Rituximab. Tocilizumab. Ruxolitinib. HSCT	IVIG. MMF. CTLA4 replacement. Abatacept. Sirolimus. Rituximab. HSCT.	Steroids. IVIG. Abatacept. Sirolimus. Rituximab. HSCT	Steroids, immunosuppressive drugs. IVIG. Rituximab. HSCT

	T regs defects				Other ALPS-like related genes			
	CD122		DEF6		TET2	TPP2	STAT1	IL12RB1
**IUIS classification**	Immune dysregulation		Immune dysregulation		Not published	Immune dysregulation	Innate immunity defect	Innate Immunity defect
**Inheritance**	AR		AR		AR	AR	AD GOF	AR
**Clinical features**								
Lymphoproliferation	S, H, L		EBV-LPD. S, H, L		S, H, L	S, L	S, H, L	S, H, L
Autoimmunity	Cytopenia, AIE, vasculitis		Cytopenia		Cytopenia, AIT	Cytopenia	Cytopenia, SLE, AIT, hepatitis	Cytopenia
Malignancy	NR		HL*		HL, NHL	NR	NR	NR
Associated features	RI, skin lesions, diarrhea, hepatitis		RI, IBD, cardiomyopathy, arthritis		RI, liver dysfunction	RI, skin lesions	CMC, RI, skin lesions, diarrhea, lung disease	Recurrent leishmaniasis, MSMD, adenitis, skin lesions, vasculitis
**ALPS parameters**								
DNT	NR		NR		High	Normal	NR	Normal / High
Vitamin B12	NR		NR		NR	Normal	NR	High
sFASL	NR		NR		Moderate high	Normal	NR	Low
IL-10	NR		NR		High	NR	NR	High
IgG level	High		Normal		High	High	Normal/High	High
IgA level	High		Normal		Low	Normal	Normal/High	Normal/High
IgM level	High		Normal		Low	High	Low/Normal	Normal/High
**Immunophenotype**								
T-cell compartment	Low/Normal CD8. Low Tregs		Inverted CD4:CD8 ratio. Low CD4 naïve. Low/ Normal/high Tregs		Low TFH	Low CD4 naïve. High senescence	Inverted CD4:CD8 ratio. Low T naïve. High Th17	Normal
B-cell compartment	Normal		Normal		Low BSM	B lymphopenia. High senescence	Low BSM	B lymphopenia
NK-cell compartment	High NK		Normal		Normal	Normal	Normal	Normal
Other	NR		NR		NR	NR	NR	NR
**Clinical functional assays**	Impaired T proliferation. Low CD122 cell surface expression.		Low DEF6 expression. CTLA4 cycling defect.		Low TET2 expression and enzymatic activity. Poor vaccine response	Low TPP2 expression. Impaired CD8 proliferation	Decreased IL-17 production. Poor vaccine response	Low IL12RB1, FASL expression and soluble FASL
**Research functional assays**	Low STAT3 and STAT5 phosphorylation		Low ERK and AKT phosphorylation		DNA hypermethylation	–	Normal/High STAT1 phosphorylation (**Figure 6D**).	Low STAT4 phosphorylation
**Treatment**	Rituximab. Sirolimus. HSCT		Abatacept		Steroids, IVIG. Rituximab. HSCT	Steroids. IVIG. MMF. Rituximab. Sirolimus. HSCT	Steroids. Ruxolitinib. sulbactam + itraconazole. HSCT	Steroids. Liposomal amphotericin B. Miltefosine. Meglumine antimoniate.

	Other ALPS-like related genes							
	ADA2		TNFAIP3		NRAS / KRAS (RALD)		CARD11	
**IUIS classification**	Autoinflammation		Autoinflammation		Phenocopy		Antibody deficiencies	
**Inheritance**	AR		AD LOF		Somatic		AD GOF	
**Clinical features**								
Lymphoproliferation	S, H, L		S, H, L		S, H, L		S, L	
Autoimmunity	Cytopenia		Cytopenia		Cytopenia		Cytopenia	
Malignancy	NR		NR		B-cell lymphoma		B-CLL	
Associated features	RI, skin lesions, neurological affection, stomatitis, arthritis, vasculitis		RI, skin lesions, diarrhea, liver dysfunction, ulcers, arthritis		Arthralgias, pericarditis, IBD, skin lesions		RI, skin lesions, HLH	
**ALPS parameters**								
DNT	Normal / High		Normal / High		Normal/High		Normal/ High	
Vitamin B12	Moderate High		Normal		NR		NR	
sFASL	NR		High		Normal/High		NR	
IL-10	High		High		Normal/High		NR	
IgG level	Normal		Normal/ High		Normal/High		Normal/High	
IgA level	Normal		Low/ High		Normal/High		Low/Normal	
IgM level	Low/ Normal		Normal/ High		Normal/High		Normal	
**Immunophenotype**								
T-cell compartment	High CD4 naïve, low CD4 memory. Low TFH		Low T naïve, high T memory. Low Th1		Normal		Normal/ Moderate T lymphopenia	
B-cell compartment	Normal / High B naïve, low BM		Low/normal BSM		B lymphocytosis		B lymphocytosis. Low BM and BSM	
NK-cell compartment	Normal		Low/normal NK		Normal		Normal	
Other	NR		NR		Monocytosis		NR	
**Clinical functional assays**	–		Low TNFAIP3 expression.		–		Poor vaccine response	
**Research functional assays**	Low ADA2 enzymatic activity		High NF-κB signaling		–		Constitutive NF-κB activation	
**Treatment**	Steroids. IVIG. MMF. Sirolimus. Etanercept. HSCT		Steroids. IVIG. Anakinra.		Steroids. IVIG. Sirolimus. Rapamycin. Hydroxychloroquine.		Steroids. Sirolimus. IVIG. Rituximab. HSCT	

### ALPS-Like Clinical Phenotype: Beyond Autoimmunity and Lymphoproliferation

According to the working definitions for clinical diagnosis of ALPS from the ESID (35), all ALPS-like cases included in this manuscript presented with lymphadenopathies and splenomegaly or hepatomegaly or all three. Immune-mediated cytopenia were also present in all patients, being the autoimmune hemolytic anemia (AIHA), immune thrombocytopenic purpura (ITP), autoimmune neutropenia (AN) or multilineage cytopenia the most common manifestations of autoimmunity (**Table 1**), as previously described in other patients with human IEI (33, 36–38). Unlike ALPS, organ-specific autoimmunity was commonly observed in ALPS-like patients, such as autoimmune thyroiditis in PIK3R1 or STAT1 GOF patients (39, 40), autoimmune polyarthritis in STK4 patients (22) autoimmune enteropathy in XIAP, STAT3 GOF, CTLA4, LRBA and IL2RB patients (16, 41–46) or autoimmune hepatitis in STAT1 GOF or LRBA patients (40, 47) **(** **Table 1** **).**

The lymphoproliferation and the immune dysregulation predispose both ALPS and ALPS-like patients to malignancies, especially lymphoma or leukemia (48, 49). In the ALPS-like group with EBV-susceptibility, several patients developed an EBV-driven B-cell lymphoma from uncontrolled EBV infection (23, 50–54). No ALPS-like patient with a defect in the *PRKCD*, *XIAP*, *IL2RA*, *IL2RB*, *TPP2*, *STAT1* GOF, *IL12RB1*, *ADA2* and *TNFAIP3* genes presented malignancy in the reviewed literature (**Table 1**).

**Figure 2:**
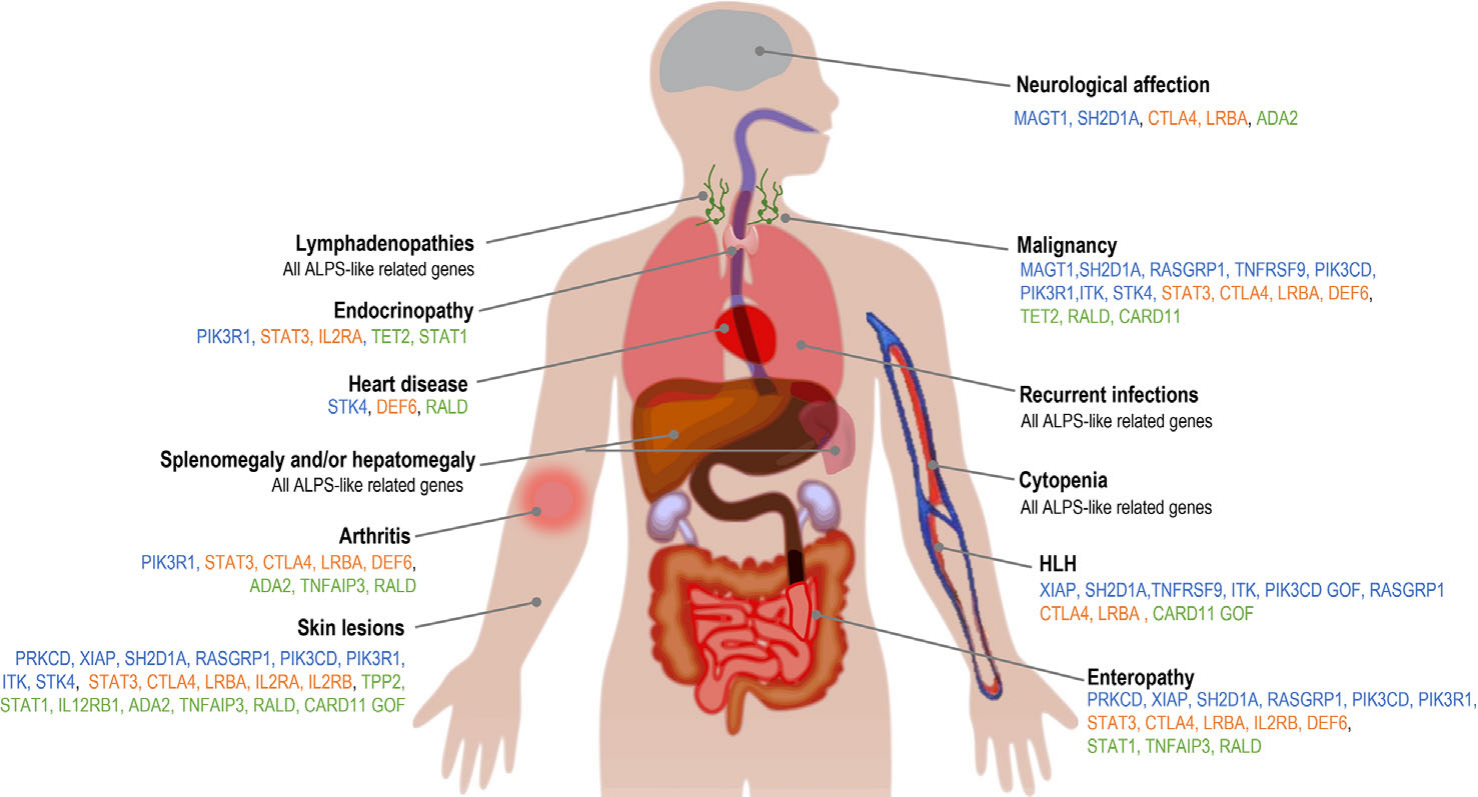
Clinical features associated with ALPS-like disorders. Blue: ALPS-like related genes with EBV-susceptibility; orange: ALPS-like related genes with regulatory T-cells defect; green: other ALPS-like related genes.

Apart from the clinical conditions shared with ALPS, phenotypic presentation of ALPS-like disorders is highly heterogeneous (**Figure 2** **)**. Recurrent infections are the most common condition associated to ALPS-like patients, in contrast to ALPS, where very few cases with immunodeficiency have been reported (13, 14, 29). Lower and upper respiratory tract infections, both viral and bacterial, were presented in most ALPS-like patients with Tregs defect, ADA2 and CARD11 GOF patients (46, 47, 55–59). Less frequently, candida or recurrent herpes zoster infections were also found in STAT3 GOF, CTLA4 and LRBA patients (46, 60). Susceptibility to predominantly viral infections was observed in TET2, TPP2 and TNFAIP3 patients (27, 61, 62) while chronic mucocutaneous candidiasis (CMC) and mendelian susceptibility to mycobacterial disease (MSMD) were characteristic of STAT1 GOF and IL12RB1 patients, respectively (40, 63).

The gastrointestinal involvement, both autoimmune or inflammatory, was described in many of these patients with immune dysregulation and ALPS-like phenotype (**Figure 2** and **Table 1** **)**. Inflammatory bowel disease (IBD) was associated to a greater extent with ALPS-like patients with regulatory T-cells defect (**Table 1** **)** and specifically the early-onset IBD turned out to be characteristic of LRBA patients compared to others (47, 64–66).

Hemophagocytic lymphohistiocytosis (HLH) is a criterion of severity associated with significant mortality. ALPS-like patients who have developed HLH should undergo HSCT. HLH turned out to be characteristic of patients with X-linked lymphoproliferative syndrome (XIAP and SH2D1A patients) and was also found in ten patients with TNFRSF9 (51), LRBA (67), ITK (53, 68), RASGRP1 (69) deficiencies, CTLA4 haploinsufficiency (55, 70), CARD11 GOF (71) and PIK3CD GOF (52) (**Table 1** **)**.

Skin involvement was remarkable in TNFAIP3 and ADA2 deficiencies, but it was one of the most common manifestations among ALPS-like disorders (**Figure 2** **),** mainly in form of non-infectious skin rashes, psoriatic-like lesions, skin abscess, severe eczema and skin warts (usually due to papilloma virus infection). Candida infection of the skin was characteristic of STAT1 GOF patients. Less frequently, arthritis, neurological affection (with both central and peripheral nervous system implication), cardiomyopathy and endocrinopathy were other clinical complications **(** **Figure 2** and **Table 1** **).**

Thus, in a suspected ALPS patient presenting with clinical complications beyond lymphoproliferation and immune-mediated cytopenia, an ALPS-like syndrome should be considered.

### Laboratory Features of ALPS-Suspected Patients: Immunophenotyping, ALPS Biomarkers and Functional Assays

There are no clinical or laboratory guidelines for the diagnosis of ALPS-like patients. Immunophenotyping of DNT by flow cytometry and typical ALPS biomarkers should be testing in any suspected ALPS patient (29, 72). A recent study established that 51.5% of probably ALPS-like patients presented abnormally high DNT, meaning that is not pathognomonic in this type of syndromes (73).

**Figure 3:**
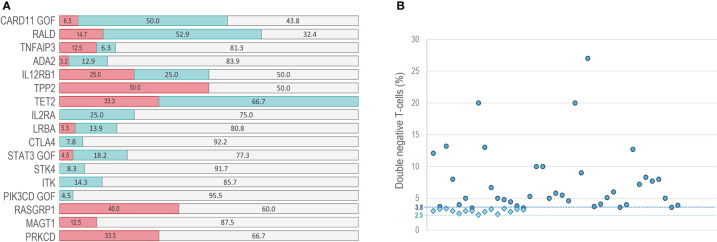
DNT in ALPS-like patients. **(A)** percentage of patients with normal (red bars) increased (blue bars) or not reported (gray bars) values of DNT. **(B)** range of DNT percentage presented in patients with high DNT. Circles: percentage of DNT in CD3+TCRαβ+ lymphocytes. Rhombus: percentage of DNT in CD3+ lymphocytes. Dashed line at point 3.8: DNT cutoff in CD3+TCRαβ+ lymphocytes. Dashed line at point 2.5: DNT cutoff in CD3+lymphocytes.

Although ALPS parameters are not provided in most of reviewed literature, increased DNT values have been reported in some ALPS-like patients with a defect in *PIK3CD* GOF (74)*, ITK* (19)*, STK4* (22)*, STAT3* GOF (24, 43, 46, 75, 76)*, CTLA4* (55, 60)*, LRBA* (16, 20, 47, 67, 77–81)*, IL2RA* (82)*, TET2* (27)*, IL12RB1 (* 26, 63)*, ADA2* (28, 58, 83, 84)*, TNFAIP3* (62, 85)*, NRAS/KRAS* (86–89) and *CARD11* GOF (59, 71, 90, 91) genes, while DNT values within the normal range were observed in PRKCD (92, 93), MAGT1 (23, 94), RASGRP1 (69) and TPP2 (61) ALPS-like patients **(** **Table 1** and **Figure 3A** **)**. We show that elevated DNT values in ALPS-like patients were found with an average of 3.0% (2.4-3.4%) respect to CD3+ lymphocytes and 7.5% (3.5-27%) respect to CD3+TCRαβ+ lymphocytes **(** **Figure 3B** **)**. Serum levels of vitamin B12, sFASL or IL-10 were only reported in seventeen patients. Different combinations of altered parameters were found. The isolated elevation of DNT was the most common alteration among patients (**Supplementary Figure 2**).

Alteration in the B-cell compartment is a common factor in most ALPS-like disorders in the term of decreased memory B-cells and increased transitional B-cells **(** **Table 1** **)**. Inverted CD4/CD8 ratio, decreased naïve and increased memory and effector memory T-cells populations are also an extended phenotype in those ALPS-like patients with a compromised T-cells compartment (**Table 1**).

**Figure 4:**
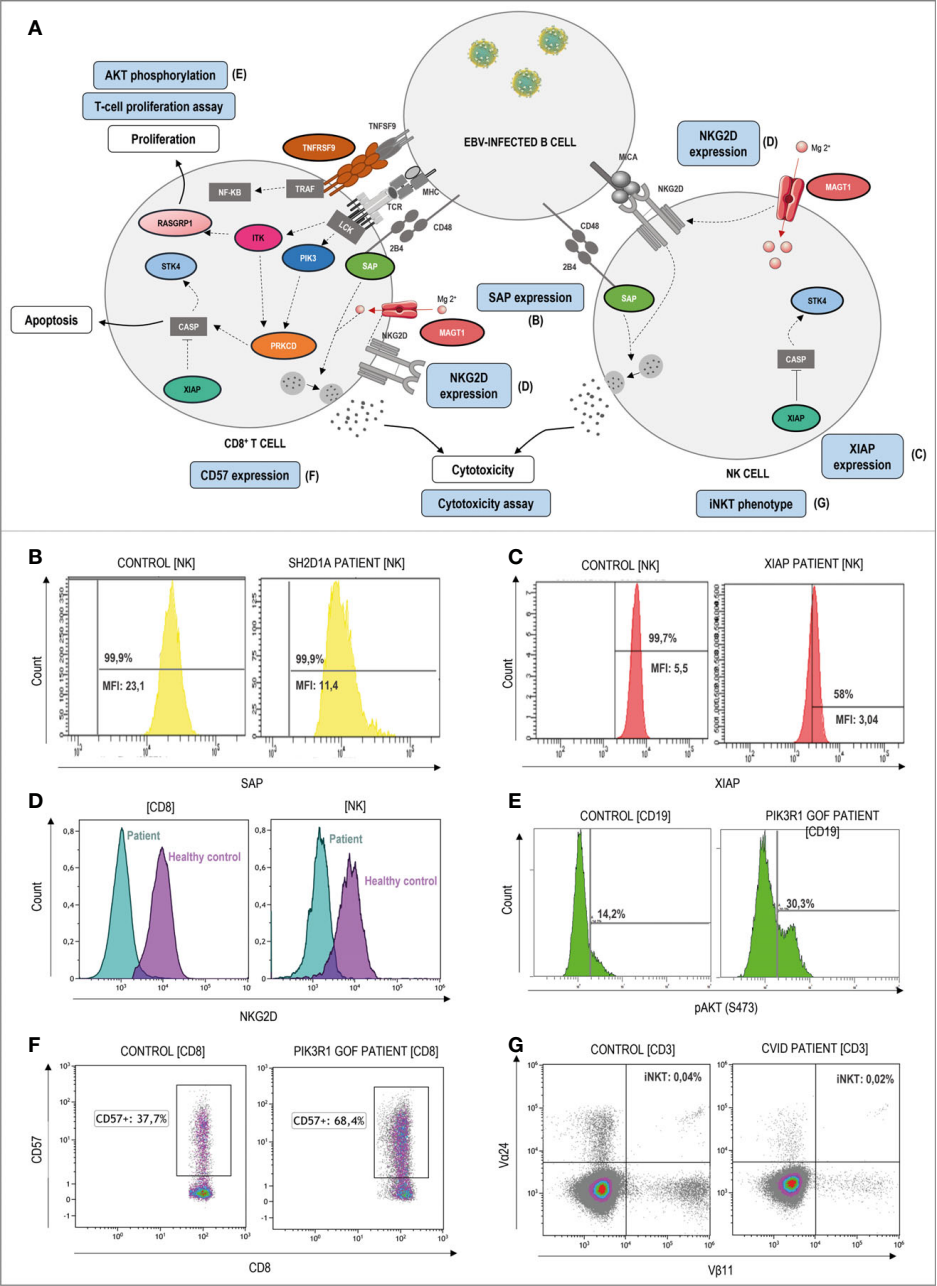
Pathophysiology and functional assays through flow cytometry in ALPS-like patients with EBV-susceptibility. **(A)** Schematic representation of the genetic defects (in color) and pathways identified in ALPS-like patients predisposing to high susceptibility to Epstein-Barr Virus (EBV)-driven lymphoproliferative disease. Immunologic studies are outlined in blue. The letters in parentheses refer to the functional studies included in figure 4. Defects in the control of EBV infection is mainly due to impairment of CD8, NK and NKT cell cytotoxicity (due to SAP, MAGT1 and TNFRSF9 deficiencies) and/or EBV-specific T cell proliferation (due to ITK, RASGRP1, TNFRSF9 deficiencies, PIK3CD gain of function and PIK3R1 loss of function) or survival (due to XIAP and STK4 deficiencies). **(B)** Decreased but not absent SAP expression in NK-cells of a patient with SAP deficiency. MFI: median fluorescence Intensity. **(C)** Decreased but not absent XIAP expression in NK-cells of a patient with XIAP deficiency. **(D)** Decreased NKG2D expression both in CD8 T and NK-cells of a MAGT1 patient (blue) in comparison with a healthy control (purple). **(E)** Hyperactivation of the PI3K-AKT signaling pathway is shown as high levels of AKT phosphorylation in a patient with PIK3R1 defect. **(F)** Senescence phenotype of CD8+CD57+ T-cells in a PIK3R1 patient. **(G)** Decreased CD3+Vα24+Vβ11+ invariant NKT-cells in a patient with common variable immunodeficiency and ALPS-like features.

**Figure 5:**
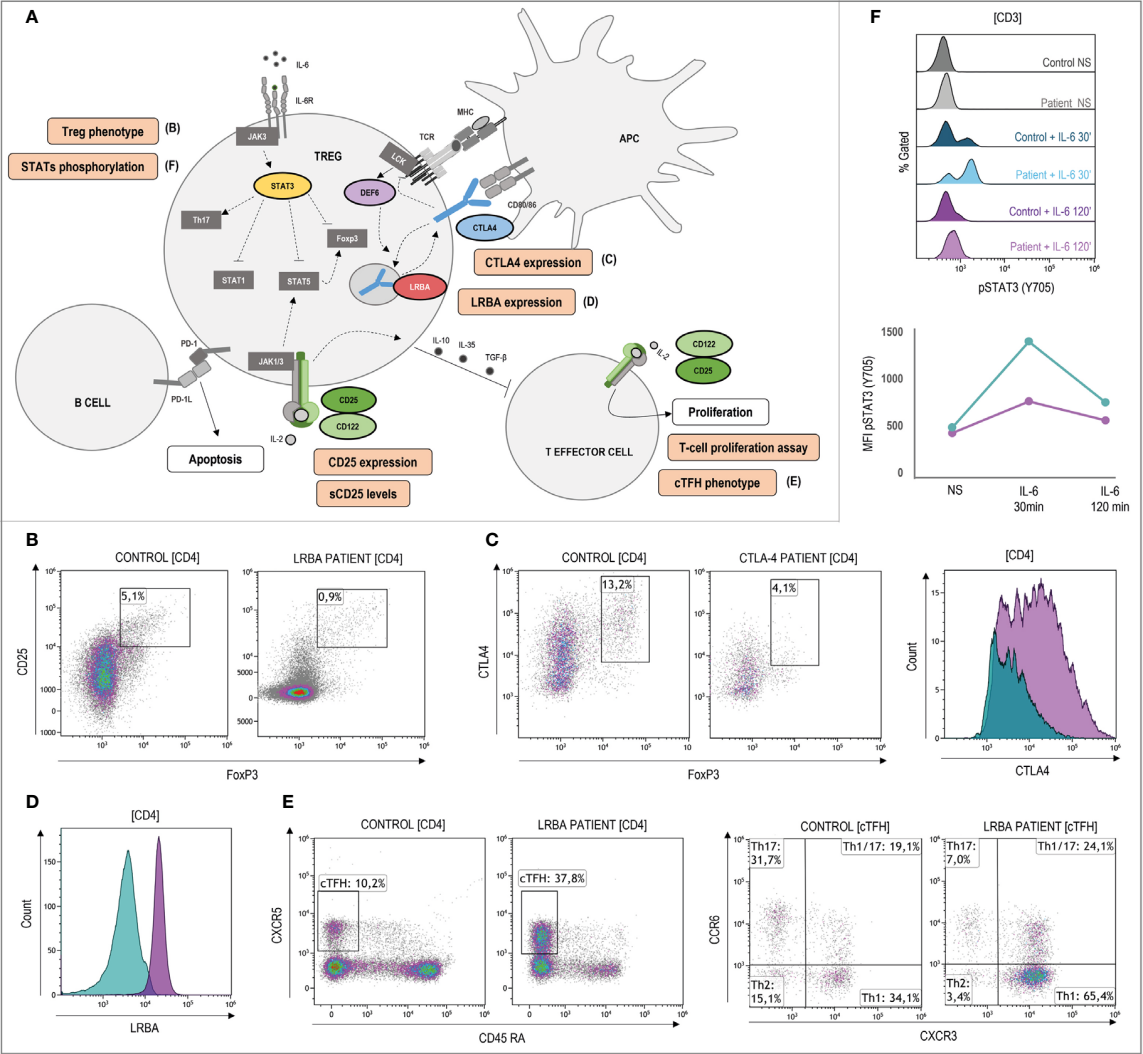
Pathophysiology and functional assays through flow cytometry in ALPS-like patients with regulatory T-cells defect. **(A)** Schematic representation of the genetic defects (in color) and pathways identified in ALPS-like patients with Treg defect. Immunologic studies are outlined in orange. The letters in parentheses refer to the functional studies included in Figure 5. Altered Tregs levels and/or function is shown in patients with CTLA4, LRBA, DEF6, IL2RA and IL2RB deficiencies and gain of function of STAT3. **(B)** Decreased CD4+CD25+FoxP3+ Treg cells in a LRBA patient. **(C)** Lower CTLA4 levels in a patient with CTLA4 haploinsufficiency (blue) in comparison with the healthy control analyzed (purple). **(D)** Decreased LRBA expression in CD4 T-cells of a patient with LRBA deficiency (blue) in comparison with the healthy control analyzed (purple). **(E)** Expansion of CD4+CXCR5+CD45RA- circulant follicular helper T cells (cTFH) and polarization to CCR6-CXCR3+ Th1 phenotype in a LRBA patient. **(F)** Hyperphosphorylation and delayed dephosphorylation of STAT3 (in tyrosine 705) induced by IL-6 in a patient with gain of function of STAT3 in comparison of the healthy control included. Line graph shows the median intensity fluorescence of STAT3 phosphorylated in the STAT3 GOF patient (blue) and the healthy control (purple). NS, no stimulation.

**Figure 6:**
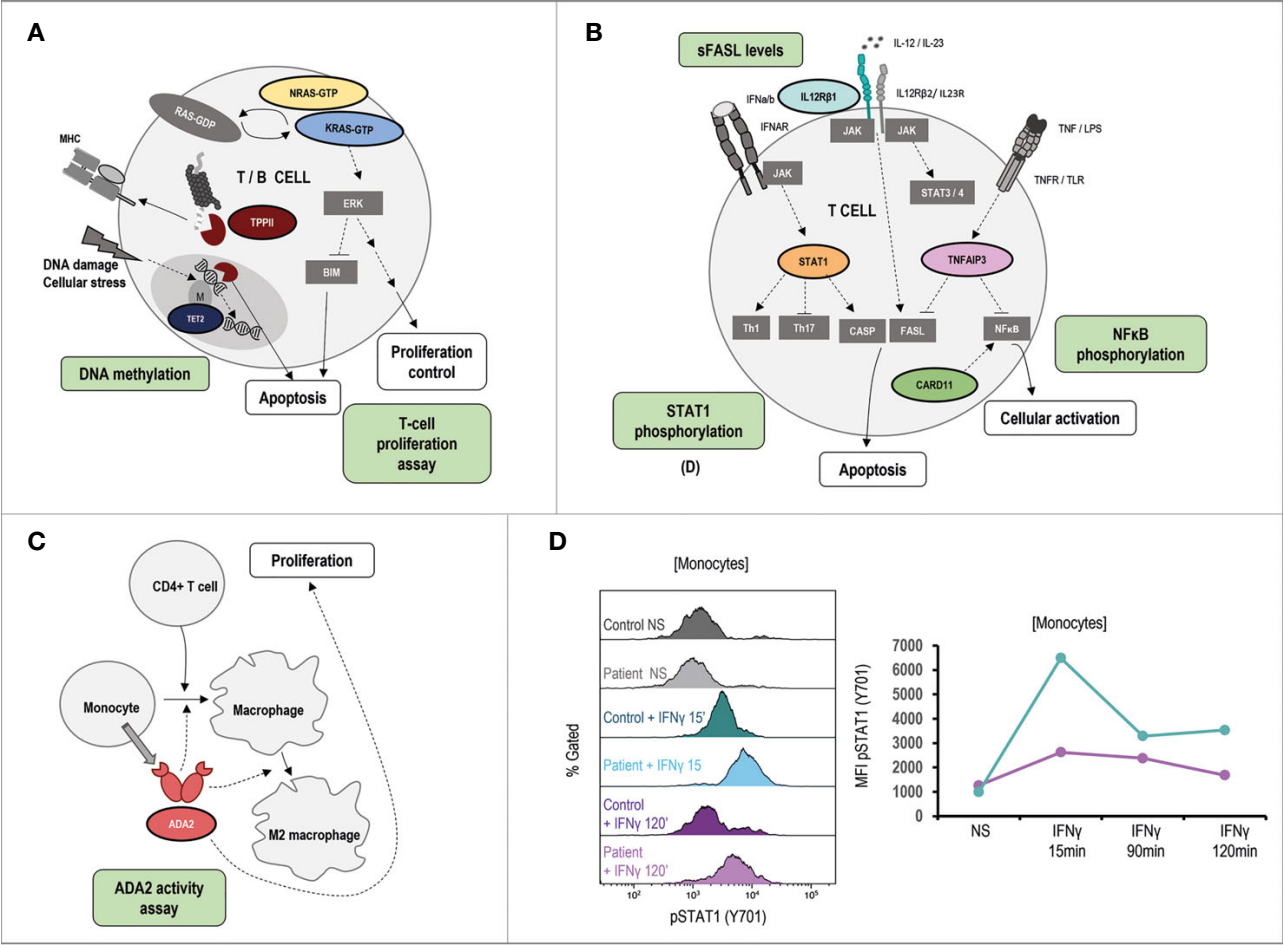
Pathophysiology and functional assays in other ALPS-like related genes. Immunologic studies are outlined in green. It is shown schematic representation of the genetic defects and pathways identified in ALPS-like patients with RALD, TTP2 and TET2 disorders **(A)**, IL12RB1, STAT1 GOF, TNFAIP3, CARD11 GOF **(B)**, ADA2 **(C)**. **(D)** Hyperphosphorylation and delayed dephosphorylation of STAT1 (in tyrosine 701) induced by IFNγ in patient´s monocytes with gain of function of STAT1 in comparison of the healthy control included. Line graph shows the MFI of STAT1 phosphorylated in the STAT1 GOF patient (blue) and the healthy control (purple). NS, no stimulation.

In those cases where the disease-causing mutation is not known or the variant is of uncertain significance (VUS), flow cytometry can be a powerful tool for the diagnosis approach through the study of extracellular and intracellular protein expression as well as the functional assessment of specific cell populations. **Figures 4**–**6** show signaling pathways and its role in the pathogenesis of ALPS-like. It is shown some helpful functional assays through flow cytometry in ALPS-like patients diagnosed in the authors’ laboratories that were not part of the PRISMA. Other functional assays reported in the literature are represented in **Table 1**.

In respect to the ALPS-like group with EBV-susceptibility (**Figure 4A** **)**, an abolished or diminished SAP and XIAP expression may be indicative of mutations in *SH2D1A* and *XIAP* genes, respectively (**Figures 4B, C** **)**. Alteration of *MAGT1* gene abolishes TCR-induced transient Mg^2+^ influx and, consequently, the expression of NKG2D receptor is decreased (**Figure 4D**). The hyperactivation of the PI3K-AKT pathway is translated in an increased AKT phosphorylation in B-cells by flow cytometry. This assay can be used to screen patients with GOF and LOF mutations in *PIK3CD* and *PI3KR1*, respectively (**Figure 4E**), which are also characterized by a senescence phenotype of CD8 T-cells with high CD57 expression (**Figure 4F**). XIAP, SAP and ITK-deficient patients have low or absent numbers of iNKT cells (see iNKT in a CVID patient with an ALPS-like phenotype in **Figure 4G**) suggesting that these genes are required for the maturation, survival and/or differentiation of NKT cells.

CTLA4, LRBA, DEF6, CD25, CD122 and STAT3 are involved in Treg function and regulation (**Figure 5A**). The low percentage of Treg detected by flow cytometry (**Figure 5B** **)** should alert of these disorders, although some patients show normal or high levels, and it is questioned if the Treg deficiency could be secondary to the immune dysregulation presented in these patients. Determination of LRBA (and sometimes CTLA4) expression levels upon T-cell stimulation can be decisive for diagnostic approaches using flow cytometry (**Figures 5C, D** **)**. In case of CTLA4, transendocytosis assay is recommendable to elucidate the result obtained by flow cytometry. CTLA4 and LRBA deficiencies show increased frequencies of circulating follicular helper T-cells (cTFH) with a polarized TH1-like phenotype (**Figure 5E**). Although it is not a fact exclusively of these genetic defects, the phenotypic characterization of TFH can be useful to rule out other ALPS-like related genes that also lead to decreased levels and/or function of Tregs. STAT3 hyperphosphorylation (**Figure 5F** **)** can explain the gain of function only in few patients whereas a normal result cannot exclude a STAT3 GOF patient, due to the high heterogeneity shown in these patients. Therefore, it is considered as a research functional assay with low diagnostic yield. In the case of CD25 deficiency, very low levels of soluble CD25 and/or CD25 expression could also be used for the diagnosis.

TET2, TPP2, STAT1 (GOF), IL12RB1, ADA2, TNFAIP3, CARD11 (GOF) and KRAS/NRAS (Ras-associated autoimmune leukoproliferative disorder, RALD) are other ALPS-like related genes (**Figures 6A–C**). As in the case of STAT3 GOF, some STAT1 GOF patients show STAT1 hyperphosphorylation (**Figure 6D**).

### Treatment, Management, and Monitoring of ALPS-Like Patients

It is recommended that patients with a probable diagnosis of ALPS be managed in the same way as patients with a definitive diagnosis (9). The first-line management of ALPS-like patients is based on treatment of the disease manifestations and complications, including lymphoproliferation, autoimmunity and infections.

Short-term corticosteroid in combination with immunosuppressive drugs as Mycophenolate (MMF) was the first-line treatment for autoimmune cytopenia and specifically AIHA in most of revised patients (**Table 1** **)**. High-dose intravenous immunoglobulin G (IVIG) therapy, together with corticosteroids was also a first-line option in case of immune thrombocytopenia. As second-line option, rituximab was used when first-line treatments failed **(** **Table 1** **)**.

Sirolimus is a mTOR inhibitor used for management of ALPS patients (95). Several ALPS-like patients with elevated DNT suffering from CTLA4 haploinsufficiency, LRBA, TPP2, ADA2 and IL2RB deficiencies, RALD syndrome or CARD11 GOF were treated and responded to this drug (31, 44, 55, 61, 71, 83, 96–98) (**Table 1** **)**.

Targeted therapies for certain molecular defects allowed patients to benefit from treatment for their underlying disease. For example, abatacept in CTLA4 haploinsufficiency, LRBA and DEF6 deficiencies; tocilizumab in STAT3 GOF; JAK inhibitors in STAT3 and STAT1 GOF patients; alemtuzumab for CD25 deficiency; PIK3δ inhibitors for APDS or anti-TNFα therapy for vasculitis in ADA2 deficiency (**Table 1** **)**. In ALPS patients, splenectomy and rituximab should be discouraged unless they are the only remaining measures to control chronic refractory life-threatening cytopenia. In case of ALPS-like patients, these decisions should be made on a case-by-case basis (29, 98).

Hematopoietic stem cell transplantation (HSCT) was carried out when the patient progressed to severe disease, as is the case of severe immunodeficiency, refractory cytopenia, HLH and high risk of malignancy. In this study we show HSCT as a possible therapeutic option in 17 of the 24 ALPS-like disorders (70.8% of genetic defects) (**Table 1**). Considering the cohort of 645 ALPS-like patients, HSCT was a therapeutic option in 108 of them (16.7%). The outcome had positive effect in 82 patients (75.9%) *vs.* negative effect in 26 (24.1%) (**Supplementary Table 4**). HSCT in ALPS-like patients is recommended in cases of SH2D1A, APDS, ADA2, and probably increasingly for LRBA. There is sufficient evidence of the potential benefit in XIAP, CTLA4, but less in STAT3, STAT1, CARD11 GOF, CD25 or CD122 deficiencies (99).

**Figure 7:**
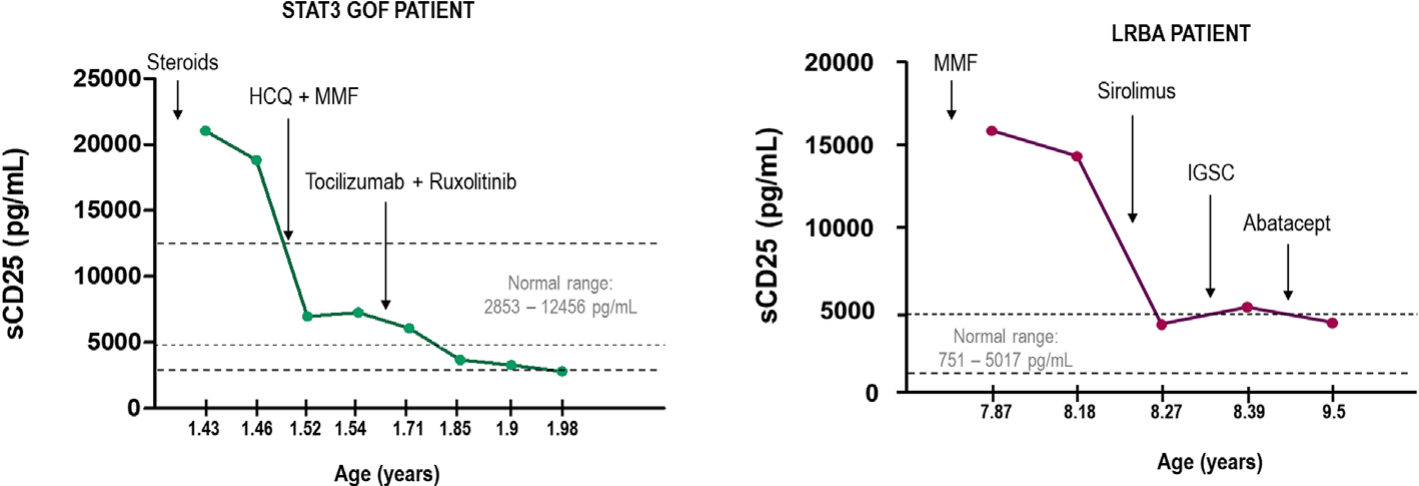
Monitorization of two cases of STAT3 GOF and LRBA deficiency measuring sCD25. Arrows indicate the changes in the treatment. HCQ, hydroxychloroquine; MMF, mycophenolate mofetil; SCIg, subcutaneous immunoglobulin therapy.

Finding the therapeutic target is as important as monitoring the prognosis of the patient and the effectiveness of the treatment. In the context of immune dysregulation, elevated serum sCD25 is indicative of chronic immune activation and it can be helpful as a clinical biomarker of the disease and treatment monitoring. Here we show two cases of LRBA deficiency and STAT3 GOF, where measuring sCD25 allowed to evaluate the course of the disease and the efficacy of the treatment (**Figure 7**). In the case of LRBA patients, sCD25 tightly correlated with other markers of immune dysregulation such as the study of cTFH (100). Thus, monitoring sCD25 and cTFH cell frequencies in LRBA and CTLA4 deficiencies maybe particularly useful in tracking disease activity and response to different therapies. Accordingly, increased frequencies of cTFH cells have been involved in the pathogenesis of several autoimmune diseases and their frequency positively correlated with serum autoantibody titers (101).

## Concluding Remarks

In recent years, multiple monogenic causes of PIRD have been identified, some of them overlap clinical and immunologic features with the well-known ALPS syndrome. The prevalence and true incidence of ALPS-like disorders is unknown, probably because many cases remain undiagnosed or misdiagnosed.

More and more genes responsible for lymphoproliferation and autoimmunity are being described. In this study, twenty-four genetic disorders with ALPS-like characteristics have been identified in the literature: PRKCD, MAGT1, XIAP, SH2D1A, RASGRP1, TNFRSF9, ITK, STK4, CTLA4, LRBA, CD25, CD122, DEF6, TET2, TPP2, IL12RB1, ADA2 and TNFAIP3 deficiencies, PIK3CD, STAT3, STAT1 and CARD11 gain of function, PIK3R1 loss of function and RALD (caused by somatic gain of function mutations in *NRAS* and *KRAS* genes). Furthermore, other genetic defects as *IKZF1* (75)*, NFKB1* (102), *RAG1* (103) or *NFKB2* (104) deficiencies have been associated with autoimmune and lymphoproliferative features in several patients cohorts.

This genetic heterogeneity makes the diagnostic approach through immunological and functional studies can be misleading. Currently, the gold standard for the diagnosis of these diseases is the performance of molecular studies by NGS, either by a targeted gene panel or by whole exome sequencing. However, flow cytometry is a quick and effective diagnostic approach tool in the case of protein expression studies and functional evaluation of different cell subpopulations, especially in those cases of VUS.

In the management of these patients, the balance between the long-term disease control and the risk of a HSCT must be considered. Targeted and individualized therapies existing for selected PIRD can solve the fine tuning of immunosuppressive treatments in patients with increased risk of infections, mitigating the possible adverse effects and saving time to offer HSCT in better circumstances.

Definitely, a negative diagnostic workup for patients with suspected ALPS should alert clinical immunologists to consider the possibility of an ALPS-like disorder. Although the heterogeneity of the phenotype makes a clinical definition difficult, these patients require a fast and precise diagnosis to offer them the best prognostic, therapeutic considerations and monitoring, including biomarkers and organ function.

## Data Availability Statement

The original contributions presented in the study are included in the article/**Supplementary Material**. Further inquiries can be directed to the corresponding author.

## Ethics Statement

The studies involving human participants were reviewed and approved by Comité Ético de Investigación Clínica, University Hospital 12 de Octubre. Written informed consent to participate in this study was provided by the participants’ legal guardian/next of kin.

## Author Contributions

ML-N and LA contributed to conception, design of the study and drafted the manuscript. ML-N developed the database, analysis, tables and figures. LG-G, DP, OC-M, NS, ND-P, RR-P, ES, JC-R, PO, JR-C and ON conducted the clinical and immunological follow-up of the patients. LG-G, JR-C, RR-P, and ON informed the patients about the study and collected the informed consents approved by the ethics committee. ML-N, RR-G, EP-A and PB-L contributed to the performance of functional tests. All authors contributed to the article and approved the submitted version.

## Funding

This work was supported by grants from Fondo de Investigación Sanitaria (FIS-PI16/2053) to LA and LG-G. The project has been co-financed with FEDER funds. ML-N was co-financed by Fondo Social Europeo, Programa Operativo de empleo juvenil (YEI).

## Conflict of Interest

The authors declare that the research was conducted in the absence of any commercial or financial relationships that could be construed as a potential conflict of interest.

## Publisher’s Note

All claims expressed in this article are solely those of the authors and do not necessarily represent those of their affiliated organizations, or those of the publisher, the editors and the reviewers. Any product that may be evaluated in this article, or claim that may be made by its manufacturer, is not guaranteed or endorsed by the publisher.

## Abbreviations

ADA2, Adenosine Deaminase 2; ALPS, Autoimmune Lymphoproliferative Syndrome; CARD11, Caspase Recruitment Domain Family Member 11; CTLA4, Cytotoxic T-Lymphocyte Associated Protein 4; DNT, Double Negative T-cells; FASL, FAS ligand; GOF, Gain of Function; IEI, Inborn Errors of Immunity; IL12RB1, Interleukin 12 Receptor Subunit Beta 1; ITK, IL2 Inducible T Cell Kinase; KRAS, KRAS Proto-Oncogene; GTPase; LRBA, LPS Responsive Beige-Like Anchor Protein; LOF, Loss of Function; MAGT1, Magnesium Transporter 1; NRAS, NRAS Proto-Oncogene; GTPase; PID, Primary Immunodeficiency; PIK3CD, Phosphatidylinositol-4,5-Bisphosphate 3-Kinase Catalytic Subunit Delta; PIK3R1, Phosphoinositide-3-Kinase Regulatory Subunit 1; PIRD, Primary Immune Regulatory Disorders; PRKCD, Protein Kinase C Delta; RASGRP1, RAS Guanyl Releasing Protein 1; SH2D1A, SH2 Domain Containing 1A; STAT1, Signal Transducer and Activator of Transcription 1; STAT3, Signal Transducer and Activator of Transcription 3; STK4, Serine/Threonine Kinase 4; TNFAIP3, TNF Alpha Induced Protein 3; UNC13D, Unc-13 Homolog D; XIAP, X-Linked Inhibitor of Apoptosis.
