# Supplementary material for: Primary Immune Regulatory Disorders With an Autoimmune Lymphoproliferative Syndrome-Like Phenotype: Immunologic Evaluation, Early Diagnosis and Management
Source: Front Immunol. 2021 Aug 10;12:671755. doi: 10.3389/fimmu.2021.671755 (PMC8382720; doi:10.3389/fimmu.2021.671755)
Supplement: Supplementary file 1 [file DataSheet_1.docx]

**SUPPLEMENTARY MATERIAL**

**METHODS**

**Flow cytometry**

Cell surface flow cytometry staining protocol was performed for the assessment of T and B-cells compartments, T follicular helper CD4 cells (TFH), invariant NKT cells (iNKT) and NKG2D receptor expression. Whole blood was incubated with the corresponding conjugated monoclonal antibodies (mAB) (listed in supplementary table 3) during 15 minutes in dark at room temperature. Cells were lysate with FACS lysing buffer (Beckman Coulter, Miami, FL, USA). After washing with PBS, cells were acquired using a Beckman Coulter Navios cytometer (Beckman Coulter, Miami, FL, USA).

Intracellular staining was performed for the study of CTLA4, LRBA, XIAP and SAP expression. PBMCS were stimulated with anti-CD3 (0,625μg/mL) (clone UCHT1**,** BD Biosciences, San Jose, USA) and anti-CD28 (0,5μg/mL) (clone KOLT-2, BD Biosciences) in case of CTLA4 or with PHA (10 μg/mL) (Sigma-Aldrich, San Luis, Misuri, EEUU) in case of LRBA. In both cases, cells were stimulated overnight at 37ºC in a humidified 5% CO2 incubator. XIAP and SAP expression were done in freshly PBMCs without stimulation. Firstly, after cell stimulation or conditioning, cell surface was staining with the corresponding conjugated mAB (supplementary table 3) during 15 minutes in dark at room temperature. Cells were then fixed and permeabilized with the Inside Stain Kit (Miltenyi Biotec, Bergisch Gladbach, Germany). After washing, cells were stained with anti-CTLA4-PE, anti-LRBA (unconjugated + secondary anti-IgG-APC), anti-XIAP (unconjugated + secondary anti-IgG-FITC) or anti-SAP (unconjugated + secondary anti-IgG-FITC) in each case during 30 minutes at 4ºC. Cellular acquisition was performed using a Beckman Coulter Navios cytometer and data were analyzed with Kaluza 2.1 software.

**Intracellular phosphorylation assay**

For STAT3, STAT1 and AKT phosphorylation, 200µL of whole blood were stimulated with IL-6 (1000 ng/mL) (Miltenyi Biotec, Madrid, Spain), IFNγ (400 U/mL, Inmukin, Horizon Pharma) and unstimulated, respectively, at indicated times at 37ºC in a humidified 5% CO_2_ incubator. Cells were then lysed and fixed with Lyse/Fix buffer and permeabilized with phosphoflow Perm Buffer III (both BD Biosciences). After washing, cells were stained with the corresponding mAB during 30 minutes at 4ºC: anti-CD3-APC (clone UCHT1, Beckman Coulter) and anti-STAT3 (pY795)-PE (clone 4/P-STAT3, BD Bioscience, Madrid, Spain) in case of STAT3 phosphorylation assay, anti-CD14-FITC and anti-STAT1 (pY701)-PerCPy5.5 for STAT1 phosphorylation assay and anti-CD19-PE (clone SJ25C1, BD Bioscience) and anti-pAKT (pS473)-APC in case of AKT phosphorylation assay. Cellular acquisition was performed using a Beckman Coulter Navios cytometer and data were analyzed with Kaluza 2.1 software.

**Serum levels of soluble CD25**

Serum sCD25 concentrations were measured using the Human IL-2 Quantikine ELISA Kit (R&D Systems, Minneapolis, MN, USA). Patients’ sera were collected in different medical visits.

**Supplementary references**

1. Barzaghi F, Minniti F, Mauro M, Bortoli M De, Balter R, Bonetti E, et al. ALPS-Like Phenotype Caused by ADA2 Deficiency Rescued by Allogeneic Hematopoietic Stem Cell Transplantation. Front Immunol [Internet]. 2019 Jan 14 [cited 2020 Sep 22];9(JAN):2767. Available from: https://www.frontiersin.org/article/10.3389/fimmu.2018.02767/full

2. Van Eyck L, Liston A, Wouters C. Mutant ADA2 in vasculopathies. N Engl J Med [Internet]. 2014 Jul 31 [cited 2021 Apr 23];371(5):480. Available from: http://www.ncbi.nlm.nih.gov/pubmed/25075848

3. Van Eyck L, Hershfield MS, Pombal D, Kelly SJ, Ganson NJ, Moens L, et al. Hematopoietic stem cell transplantation rescues the immunologic phenotype and prevents vasculopathy in patients with adenosine deaminase 2 deficiency. J Allergy Clin Immunol [Internet]. 2015 Jan 1 [cited 2021 Apr 23];135(1):283-287.e5. Available from: https://pubmed.ncbi.nlm.nih.gov/25457153/

4. Hashem H, Vatsayan A, Gupta A, Nagle K, Hershfield M, Dalal J. Successful reduced intensity hematopoietic cell transplant in a patient with deficiency of adenosine deaminase 2 [Internet]. Vol. 52, Bone Marrow Transplantation. Nature Publishing Group; 2017 [cited 2021 Apr 23]. p. 1575–6. Available from: https://pubmed.ncbi.nlm.nih.gov/28805790/

5. Van Montfrans JM, Hartman EAR, Braun KPJ, Hennekam EAM, Hak EA, Nederkoorn PJ, et al. Phenotypic variability in patients with ADA2 deficiency due to identical homozygous R169Q mutations. Rheumatol (United Kingdom) [Internet]. 2016 May 1 [cited 2021 Apr 23];55(5):902–10. Available from: https://pubmed.ncbi.nlm.nih.gov/26867732/

6. Snow AL, Xiao W, Stinson JR, Lu W, Benjamin CD, Zheng L, et al. Congenital B cell lymphocytosis explained by novel germline CARD11 mutations. J Exp Med [Internet]. 2012 Nov [cited 2021 Apr 21];209(12):2247–61. Available from: https://pubmed.ncbi.nlm.nih.gov/23129749/

7. Greil C, Roether F, La Rosée P, Grimbacher B, Duerschmied D, Warnatz K. Rescue of Cytokine Storm Due to HLH by Hemoadsorption in a CTLA4-Deficient Patient [Internet]. Vol. 37, Journal of Clinical Immunology. Springer New York LLC; 2017 [cited 2021 Apr 23]. p. 273–6. Available from: https://pubmed.ncbi.nlm.nih.gov/28265964/

8. Schwab C, Gabrysch A, Olbrich P, Patiño V, Warnatz K, Wolff D, et al. Phenotype, penetrance, and treatment of 133 cytotoxic T-lymphocyte antigen 4–insufficient subjects. J Allergy Clin Immunol [Internet]. 2018 Dec 1 [cited 2021 Apr 21];142(6):1932–46. Available from: https://pubmed.ncbi.nlm.nih.gov/29729943/

9. Slatter MA, Engelhardt KR, Burroughs LM, Arkwright PD, Nademi Z, Skoda-Smith S, et al. Hematopoietic stem cell transplantation for CTLA4 deficiency. J Allergy Clin Immunol [Internet]. 2016 Aug 1 [cited 2021 Apr 23];138(2):615-619.e1. Available from: https://pubmed.ncbi.nlm.nih.gov/27102614/

10. Mahat U, Terzioglu MK, Buhtoiarov I. CTLA4 haploinsufficiency as a predisposition to classical Hodgkin lymphoma. Pediatr Hematol Oncol [Internet]. 2020 Feb 17 [cited 2021 Apr 23];37(2):176–83. Available from: https://pubmed.ncbi.nlm.nih.gov/31971458/

11. Goudy K, Aydin D, Barzaghi F, Gambineri E, Vignoli M, Mannurita SC, et al. Human IL2RA null mutation mediates immunodeficiency with lymphoproliferation and autoimmunity. Clin Immunol [Internet]. 2013 Mar [cited 2021 Apr 23];146(3):248–61. Available from: https://pubmed.ncbi.nlm.nih.gov/23416241/

12. Zhang Z, Gothe F, Pennamen P, James JR, McDonald D, Mata CP, et al. Human interleukin-2 receptor b mutations associated with defects in immunity and peripheral tolerance. J Exp Med [Internet]. 2019 Jun 1 [cited 2020 Nov 20];216(6):1311–27. Available from: https://pubmed.ncbi.nlm.nih.gov/31040185/

13. Kiykim A, Ogulur I, Dursun E, Charbonnier LM, Nain E, Cekic S, et al. Abatacept as a Long-Term Targeted Therapy for LRBA Deficiency. J Allergy Clin Immunol Pract [Internet]. 2019 Nov 1 [cited 2021 Apr 21];7(8):2790-2800.e15. Available from: https://pubmed.ncbi.nlm.nih.gov/31238161/

14. Azizi G, Abolhassani H, Zaki-Dizaji M, Habibi S, Mohammadi H, Shaghaghi M, et al. Polyautoimmunity in Patients with LPS-Responsive Beige-Like Anchor (LRBA) Deficiency. Immunol Invest [Internet]. 2018 Jul 4 [cited 2021 Apr 22];47(5):457–67. Available from: https://pubmed.ncbi.nlm.nih.gov/29528757/

15. Kostel Bal S, Haskologlu S, Serwas NK, Islamoglu C, Aytekin C, Kendirli T, et al. Multiple Presentations of LRBA Deficiency: a Single-Center Experience. J Clin Immunol [Internet]. 2017 Nov 1 [cited 2021 Apr 21];37(8):790–800. Available from: https://pubmed.ncbi.nlm.nih.gov/28956255/

16. Cagdas D, Halaçlı SO, Tan Ç, Lo B, Çetinkaya PG, Esenboğa S, et al. A Spectrum of Clinical Findings from ALPS to CVID: Several Novel LRBA Defects. J Clin Immunol [Internet]. 2019 Oct 1 [cited 2020 Nov 26];39(7):726–38. Available from: https://pubmed.ncbi.nlm.nih.gov/31432443/

17. Gámez-Díaz L, August D, Stepensky P, Revel-Vilk S, Seidel MG, Noriko M, et al. The extended phenotype of LPS-responsive beige-like anchor protein (LRBA) deficiency. J Allergy Clin Immunol [Internet]. 2016 Jan 1 [cited 2021 Apr 19];137(1):223–30. Available from: https://pubmed.ncbi.nlm.nih.gov/26768763/

18. Okur F V., Kuskonmaz B, Cagdas D, Tezcan I, Uckan-Cetinkaya D. Bone marrow transplantation with Favorable outcome in three patients with LPS-responsive beige-like anchor (LRBA)deficiency [Internet]. Vol. 203, Clinical Immunology. Academic Press Inc.; 2019 [cited 2021 Apr 23]. p. 162–5. Available from: https://pubmed.ncbi.nlm.nih.gov/31026575/

19. Seidel MG, Hirschmugl T, Gamez-Diaz L, Schwinger W, Serwas N, Deutschmann A, et al. Long-term remission after allogeneic hematopoietic stem cell transplantation in LPS-responsive beige-like anchor (LRBA) deficiency. J Allergy Clin Immunol [Internet]. 2015 May 1 [cited 2021 Apr 23];135(5):1384-1390.e8. Available from: https://pubmed.ncbi.nlm.nih.gov/25539626/

20. Shamriz O, Shadur B, NaserEddin A, Zaidman I, Simanovsky N, Elpeleg O, et al. Respiratory manifestations in LPS-responsive beige-like anchor (LRBA) protein-deficient patients. Eur J Pediatr [Internet]. 2018 Aug 1 [cited 2021 Apr 23];177(8):1163–72. Available from: https://pubmed.ncbi.nlm.nih.gov/29777306/

21. Sharapova SO, Haapaniemi E, Sakovich IS, Rojas J, Gámez-Díaz L, Mareika YE, et al. Novel LRBA Mutation and Possible Germinal Mosaicism in a Slavic Family [Internet]. Vol. 38, Journal of Clinical Immunology. Springer New York LLC; 2018 [cited 2021 Apr 23]. p. 471–4. Available from: https://pubmed.ncbi.nlm.nih.gov/29804237/

22. Tesi B, Priftakis P, Lindgren F, Chiang SCC, Kartalis N, Löfstedt A, et al. Successful Hematopoietic Stem Cell Transplantation in a Patient with LPS-Responsive Beige-Like Anchor (LRBA) Gene Mutation. J Clin Immunol [Internet]. 2016 Jul 1 [cited 2021 Apr 23];36(5):480–9. Available from: https://pubmed.ncbi.nlm.nih.gov/27146671/

23. Cansever M, Zietara N, Chiang SCC, Ozcan A, Yilmaz E, Karakukcu M, et al. A Rare Case of Activated Phosphoinositide 3-Kinase Delta Syndrome (APDS) Presenting with Hemophagocytosis Complicated with Hodgkin Lymphoma. J Pediatr Hematol Oncol [Internet]. 2020 Mar 1 [cited 2021 Apr 19];42(2):156–9. Available from: https://pubmed.ncbi.nlm.nih.gov/31033788/

24. Elkaim E, Neven B, Bruneau J, Mitsui-Sekinaka K, Stanislas A, Heurtier L, et al. Clinical and immunologic phenotype associated with activated phosphoinositide 3-kinase δ syndrome 2: A cohort study. J Allergy Clin Immunol [Internet]. 2016 Jul 1 [cited 2021 Apr 23];138(1):210-218.e9. Available from: https://pubmed.ncbi.nlm.nih.gov/27221134/

25. Gallo V, Cirillo E, Prencipe R, Lepore A, Del Vecchio L, Scalia G, et al. Clinical, Immunological, and Functional Characterization of Six Patients with Very High IgM Levels. J Clin Med [Internet]. 2020 Mar 17 [cited 2021 Apr 23];9(3):818. Available from: https://pubmed.ncbi.nlm.nih.gov/32192142/

26. Lucas CL, Zhang Y, Venida A, Wang Y, Hughes J, McElwee J, et al. Heterozygous splice mutation in PIK3R1 causes human immunodeficiency with lymphoproliferation due to dominant activation of PI3K. J Exp Med [Internet]. 2014 [cited 2020 Nov 19];211(13):2537–47. Available from: https://pubmed.ncbi.nlm.nih.gov/25488983/

27. Somekh I, Marquardt B, Liu Y, Rohlfs M, Hollizeck S, Karakukcu M, et al. Novel Mutations in RASGRP1 are Associated with Immunodeficiency, Immune Dysregulation, and EBV-Induced Lymphoma. J Clin Immunol [Internet]. 2018 Aug 1 [cited 2021 Apr 23];38(6):699–710. Available from: https://pubmed.ncbi.nlm.nih.gov/30030704/

28. Lankester AC, Visser LFA, Hartwig NG, Bredius RGM, Gapar HB, van der Burg M, et al. Allogeneic stem cell transplantation in X-linked lymphoproliferative disease: Two cases in one family and review of the literature. Bone Marrow Transplant [Internet]. 2005 Jul [cited 2021 Apr 23];36(2):99–105. Available from: https://pubmed.ncbi.nlm.nih.gov/15908972/

29. Sun J, Ying W, Liu D, Hui X, Yu Y, Wang J, et al. Clinical and Genetic Features of 5 Chinese Patients with X-linked lymphoproliferative Syndrome. Scand J Immunol [Internet]. 2013 Nov [cited 2021 Apr 23];78(5):463–7. Available from: https://pubmed.ncbi.nlm.nih.gov/23944711/

30. Baris S, Alroqi F, Kiykim A, Karakoc-Aydiner E, Ogulur I, Ozen A, et al. Severe Early-Onset Combined Immunodeficiency due to Heterozygous Gain-of-Function Mutations in STAT1. J Clin Immunol [Internet]. 2016 Oct 1 [cited 2020 Dec 20];36(7):641–8. Available from: https://pubmed.ncbi.nlm.nih.gov/27379765/

31. Milner JD, Vogel TP, Forbes L, Ma CA, Stray-Pedersen A, Niemela JE, et al. Early-onset lymphoproliferation and autoimmunity caused by germline STAT3 gain-of-function mutations. Blood [Internet]. 2015 Jan 22 [cited 2020 Sep 22];125(4):591–9. Available from: https://pubmed.ncbi.nlm.nih.gov/25359994/

32. Schipp C, Schlütermann D, Hönscheid A, Nabhani S, Höll J, Oommen PT, et al. EBV negative lymphoma and autoimmune lymphoproliferative syndrome like phenotype extend the clinical spectrum of primary immunodeficiency caused by STK4 deficiency. Front Immunol [Internet]. 2018 Oct 16 [cited 2020 Sep 22];9(OCT). Available from: https://pubmed.ncbi.nlm.nih.gov/30386345/

33. Stremenova Spegarova J, Lawless D, Mohamad SMB, Engelhardt KR, Doody G, Shrimpton J, et al. Germline TET2 loss of function causes childhood immunodeficiency and lymphoma. Blood. 2020 Aug 27;136(9):1055–66.

34. Alosaimi MF, Hoenig M, Jaber F, Platt CD, Jones J, Wallace J, et al. Immunodeficiency and EBV-induced lymphoproliferation caused by 4-1BB deficiency. J Allergy Clin Immunol [Internet]. 2019 Aug 1 [cited 2020 Nov 19];144(2):574-583.e5. Available from: https://pubmed.ncbi.nlm.nih.gov/30872117/

35. Stepensky P, Rensing-Ehl A, Gather R, Revel-Vilk S, Fischer U, Nabhani S, et al. Early-onset Evans syndrome, immunodeficiency, and premature immunosenescence associated with tripeptidyl-peptidase II deficiency. In: Blood [Internet]. American Society of Hematology; 2015 [cited 2020 Nov 24]. p. 753–61. Available from: https://pubmed.ncbi.nlm.nih.gov/25414442/

36. Chellapandian D, Krueger J, Schechter T, Gassas A, Weitzman S, Naqvi A, et al. Successful Allogeneic Hematopoietic Stem Cell Transplantation in XIAP Deficiency Using Reduced-Intensity Conditioning. Pediatr Blood Cancer [Internet]. 2016 Feb 1 [cited 2021 Apr 23];63(2):355–7. Available from: https://pubmed.ncbi.nlm.nih.gov/26398727/

37. Girardelli M, Arrigo S, Barabino A, Loganes C, Morreale G, Crovella S, et al. The diagnostic challenge of very early-onset enterocolitis in an infant with XIAP deficiency. BMC Pediatr [Internet]. 2015 Dec 15 [cited 2021 Apr 19];15(1). Available from: https://pubmed.ncbi.nlm.nih.gov/26671016/

38. Jiang MY, Guo X, Sun SW, Li Q, Zhu YP. Successful allogeneic hematopoietic stem cell transplantation in a boy with X-linked inhibitor of apoptosis deficiency presenting with hemophagocytic lymphohistiocytosis: A case report. Exp Ther Med [Internet]. 2016 Sep 1 [cited 2021 Apr 23];12(3):1341–4. Available from: https://pubmed.ncbi.nlm.nih.gov/27602064/

39. Lekbua A, Ouahed J, O’Connell AE, Kahn SA, Goldsmith JD, Imamura T, et al. Risk-factors Associated with Poor Outcomes in VEO-IBD Secondary to XIAP Deficiency: A Case Report and Literature Review. J Pediatr Gastroenterol Nutr [Internet]. 2019 Jul 1 [cited 2021 Apr 23];69(1):E13–8. Available from: https://pubmed.ncbi.nlm.nih.gov/31232887/

40. Marsh RA, Madden L, Kitchen BJ, Mody R, McClimon B, Jordan MB, et al. XIAP deficiency: A unique primary immunodeficiency best classified as X-linked familial hemophagocytic lymphohistiocytosis and not as X-linked lymphoproliferative disease. Blood [Internet]. 2010 Aug 19 [cited 2021 Apr 23];116(7):1079–82. Available from: https://pubmed.ncbi.nlm.nih.gov/20489057/

41. O’Rafferty C, Velangi M, Lawson S, Hiwarkar P, Motwani J. IFNɣ Block, Treosulfan Conditioning and αβ T Cell Deplete PBSCT for XIAP-Deficient HLH [Internet]. Vol. 37, Journal of Clinical Immunology. Springer New York LLC; 2017 [cited 2021 Apr 23]. p. 511–3. Available from: https://pubmed.ncbi.nlm.nih.gov/28639166/

42. Schmid JP, Canioni D, Moshous D, Touzot F, Mahlaoui N, Hauck F, et al. Clinical similarities and differences of patients with X-linked lymphoproliferative syndrome type 1 (XLP-1/SAP deficiency) versus type 2 (XLP-2/XIAP deficiency). Blood [Internet]. 2011 Feb 3 [cited 2021 Apr 23];117(5):1522–9. Available from: https://pubmed.ncbi.nlm.nih.gov/21119115/

43. Wada T, Kanegane H, Ohta K, Katoh F, Imamura T, Nakazawa Y, et al. Sustained elevation of serum interleukin-18 and its association with hemophagocytic lymphohistiocytosis in XIAP deficiency. Cytokine [Internet]. 2014 Jan [cited 2021 Apr 23];65(1):74–8. Available from: https://pubmed.ncbi.nlm.nih.gov/24084330/

44. Worth AJJ, Nikolajeva O, Chiesa R, Rao K, Veys P, Amrolia PJ. Successful stem cell transplant with antibody-based conditioning for XIAP deficiency with refractory hemophagocytic lymphohistiocytosis [Internet]. Vol. 121, Blood. American Society of Hematology; 2013 [cited 2021 Apr 23]. p. 4966–8. Available from: https://pubmed.ncbi.nlm.nih.gov/23766462/

**Supplementary figure legend**

**Supplementary figure 1.** PRISMA flow diagram. PRISMA, Preferred Reporting Items for Systematic Reviews and Meta-analyses. WoS, Web of Science

**Supplementary figure 2.** Venn diagram showing the overlap of the ALPS parameters (DNT, vitamin B12, sFASL and IL-10) in ALPS-like patients. Numbers in the individual sections represent the number of patients with alteration of each condition/s.

**Supplementary table 1.** **Search and filter publications reporting ALPS-like cases**

|  | **IUIS classification** | **Gene** | **Publications** | **Reported cases** | **Filtered publications** | **Filtered cases** |
| --- | --- | --- | --- | --- | --- | --- |
| **ALPS-like with EBV susceptibility** | **ID** | **PRKCD** | 4 | 6 | 4 | 6 |
|  | **ID** | **MAGT1** | 11 | 28 | 5 | 8 |
|  | **ID** | **XIAP** | 55 | 207 | 27 | 64 |
|  | **ID** | **SH2D1A** | 24 | 195 | 9 | 25 |
|  | **ID** | **RASGRP1** | 5 | 9 | 3 | 5 |
|  | **ID** | **TNFRSF9** | 2 | 6 | 2 | 3 |
|  | **AD** | **PIK3CD GOF** | 23 | 187 | 10 | 22 |
|  | **AD** | **PIK3R1 LOF** | 18 | 69 | 5 | 11 |
|  | **CID** | **ITK** | 14 | 30 | 4 | 7 |
|  | **CID** | **STK4** | 9 | 18 | 6 | 12 |
| **ALPS-like with regulatory T-cells defect** | **ID** | **STAT3 GOF** | 22 | 72 | 13 | 44 |
|  | **ID** | **CTLA4** | 26 | 154 | 26 | 154 |
|  | **ID** | **LRBA** | 56 | 270 | 39 | 151 |
|  | **ID** | **CD25** | 7 | 7 | 4 | 4 |
|  | **ID** | **CD122** | 2 | 10 | 1 | 4 |
|  | **ID** | **DEF6** | 2 | 7 | 2 | 6 |
| **Other ALPS-like related genes** | **AI** | **TET2** | 1 | 3 | 1 | 3 |
|  | **ID** | **TPP2** | 2 | 6 | 1 | 2 |
|  | **IID** | **STAT1 GOF** | 71 | 334 | 9 | 13 |
|  | **IID** | **IL12RB1** | 59 | 186 | 4 | 4 |
|  | **AI** | **ADA2** | 65 | 272 | 12 | 31 |
|  | **AI** | **TNFAIP3** | 25 | 105 | 8 | 16 |
|  | **Phenocopy** | **NRAS/KRAS** | 13 | 34 | 13 | 34 |
|  | **AD** | **CARD11 GOF** | 10 | 30 | 6 | 16 |
| **Total** |  | **24** | **526** | **2245** | **214*** | **645** |

*References of all publications used in the meta-analysis are shown in supplementary table 2. *Abbreviations: AD: antibody deficiency; AI: autoinflammation; CID: combined immunodeficiency; ID: immune dysregulation; IID: innate immunity defect;*

**Supplementary table 2. References used in the meta-analysis**

|  | **REFERENCES** |  |
| --- | --- | --- |
| Abdollahpour H Blood 2012 | Elkaim E J Allergy Clin Immunol 2016 | Kracker S J Allergy Clin Immunol 2014 |
| Al Sukaiti Clin Transl Immunology 2017 | Erdős M Front Immunol 2020 | Kucuk ZY J Allergy Clin Immunol 2017 |
| Alangari A J Allergy Clin Immunol 2012 | Eren Akarcan S JMM Case Rep 2018 | Kuehn HS Blood 2013 |
| Alkhairy OK J Clin Immunol 2016 | Fabre A AJRCCM 2018 | Kuehn HS Science 2014 |
| Alosaimi MF J Allergu Clin Immunol 2019 | Fernandez KS Pediatr Blood Cancer 2019 | Kutluǧ Ş Centr Eur J Immunol 2019 |
| Al-Saud B J Clin Immunol 2019 | Fournier B J Allergy Clin Immunol 2020 | Kwon WK Allergy Asthma Immunol Res 2020 |
| Alsultan A Pediatr Blood Cancer 2018 | Franco-Jarava C Clin Immunol. 2018 | Lai N J Allergy Clin Immunol Pract 2019 |
| Ammann S Clin and Exp Immunol 2014 | Gabrysch A J Allergy Clin Immunol 2017 | Lankester AC Bone Marrow Transplant 2005 |
| Ayrignac X Neurology 2020 | Gamez-Diaz J Allergy Clin Immunol 2016 | Lau CY AnnalsATS 2016 |
| Azizi G Immunol Invest. 2018 | Ganhão S Clin Rheumatol 2020 | Lekbua A J Pediatr Gastroenterol Nutr 2019 |
| Azizi G J Investig Allergol Clin Immunol 2018 | Gans MD J Clin Immunol 2020 | Lévy E Clin Immunol 2016 |
| Azizi G PAI 2017 | García-Morato MB Clin Immunol 2017 | Levy-Mendelovich S Clin Exp Immunol 2017 |
| Bal SK J Clin Immunol 2017 | Gayo V J. Clin. Med. 2020 | Li FY Blood 2014 |
| Baris S J Clin Immunol 2016 | Ghaini M Immunol Invest 2020 | Li G Medicine (Baltimore) 2020 |
| Baroudi SA BMJ Case Rep 2019 | Giacaman A Clin Exp Dermatol 2018 | Li GM BMC Med Genet 2019 |
| Barzaghi F Front Immunol 2019 | Giardino G Pediatr Allergy Immunol. 2015 | Li GM Medicine 2019 |
| Becerra JCA Front Pediatr 2017 | Girardelli M BMC Pediatrics 2015 | Li R Biomed Res Int 2018 |
| Belot A Arth Rheum 2013 | Göktürk B Turk J Pediatr 2016 | Linka RM Nature 2015 |
| Ben-Ami T J Pediatr 2016 | Goudy K Clin Immunol 2013 | Liu X BMC Med Genet 2018 |
| Besnard C Clin Immunol 2018 | Greil C J Clin Immunol 2017 | Lo B Science 2015 |
| Booth C Blood 2011 | Guo X Medicine 2018 | López-Herrera G Am J Hum Genet 2012 |
| Bras J N Engl J Med 2014 | Gupta M Front Immunol 2018 | Lucas CL JEM 2014 |
| Brohl AS J Clin Immunol 2015 | Haapaniemi EM Blood 2015 | Lucas CL Nature 2013 |
| Buchbinder D J Allergy Clin Immunol 2015 | Halacli SO Clin Immunol 2015 | Maffucci P Front Immunol 2016 |
| Buchbinder D J Ped Hematol 2018 | Hashem H Bone Marrow Transplant 2017 | Maggiore R J Pediatr Hematol Oncol 2019 |
| Cagdas D J Clin Immunol 2019 | Hayakawa S J Clin Immunol 2016 | Mahat U Ped Hem and Onc 2019 |
| Calvo KR Blood 2015 | He TY Zhonghua Er Ke Za Zhi 2017 | Makadia P J Allergi Clin Immunol Pract 2017 |
| Cansever M J Pediatr Hematol Oncol 2020 | Hong Y Frontiers 2019 | Mao H J Allergy Clin Immunol 2018 |
| Cárdenes M J Med Genet 2010 | Hou TZ Clin Immunol 2018 | Marsh RA Blood 2012 |
| Caudy AA J Allergy Clin Immunol 2007 | Hoyos-Bachiloglu L J Clin Immunol 2020 | Maschan M Pediatr Blood Cancer 2014 |
| Chellapandian D Pediatr Blood Cancer 2016 | Huck K JCI 2009 | Masiaga ML J Clin Rheumatol 2015 |
| Chen X J Clin Immunol 2020 | Hügle B Eur J Pediatr 2007 | Meshaal S J Clin Immunol 2020 |
| Chiriaco M Clin Immunol 2017 | Inoue K Transpl Infect Dis 2020 | Milner JD Blood 2015 |
| Crequer A PLOS one 2012 | Jägle S Clin Immunol 2020 | Montfrans JV Pediatric Rheumatology 2015 |
| Desjardins M Front Immunol. 2018 | Jiang MY Exp Ther Med 2016 | Moraes-Fontes MF Clinical Case Reports 2017 |
| Dhalla F J Clin Immunol 2015 | Jin YY Hum Immunol 2016 | Moriya K Int J Hematol 2020 |
| Dimitrova D J Clin Immunol 2019 | Johnson and De Franco Diabetes 2017 | Morra M Blood 2001 |
| Dong X J Clin Immunol 2019 | Kadowaki T J Allergy Clin Immunol 2018 | Mozdarani H Int J Radiat Biol 2019 |
| Dulau Florea AE J Allergy Clin Immunol 2017 | Khoury T Clinical TherapeuticS 2017 | Nabhani S Clin Immunol 2017 |
| Dziadzio M J Clin Immunol 2015 | Kiykim A J Allergy Clin Immunol Pract 2019 | Nabhani S Haematologica 2015 |
| Eick Jr LV J Allergy Clin Immunol 2015 | Kiykim A J Clin Immunol 2015 | Nehme NT Blood 2012 |
| Elgizouli M ClinExp Immunol 2015 | Kolcava J Multiple Sclerosis and Related Disorders 2020 | Niemela JE Blood 2011 |

**Supplementary table 2 (continued)**

| **REFERENCES** | |
| --- | --- |
| Nistala K Clin Exp Immunol 2001 | Somekh I Blood 2019 |
| ÖF Beser JPGN 2016 | Somekh I J Clin Immunol 2018 |
| Okur FV Clin Immunol 2019 | Spegarova JS Blood 2020 |
| O'Rafferty C J Clin Immunol 2017 | Steele CL J Clin Immunol 2016 |
| Outinen T Clin Immunol 2016 | Stepensky P Blood 2015 |
| Parackova Z Cell Detah and Disease 2020 | Stepensky P Haematologica 2011 |
| Parvaneh N Pediatr Blood Cancer 2017 | Suh YH Ophthalmic Genet 2019 |
| Patiroglu T Clin Immunol 2015 | Sun J Scand J Immunol 2013 |
| Phan ANL Mol Gen and Gen Med 2020 | Sun J Scandinavian Journal of Immunology, 2013 |
| Platt CD Clin Immunol 2017 | Sundin M J Pediatr Hematol Oncol 2019 |
| Ragotte RJ Clin Immunol 2017 | Takagi M Blood 2011 |
| Rajpurkar M J Pediatr Hematol Oncol 2019 | Takagi M J Allergy Clin Immunol 2017 |
| Revel-Vil Clin Immunol 2015 | Takagi M Ped Blood and Cancer 2018 |
| Salami F End, Met & ImmDisorders 2020 | Takeda AJ J Allergy Clin Immunol 2017 |
| Salzer E Blood 2013 | Tesi B J Clin Immunol 2016 |
| Salzer U Clin Immunol 2018 | Toyoda H J Pediatr Hematol Oncol 2018 |
| Schepp J Arthritis Rheumatol 2017 | Trotta L J Allergy Clin Immunol 2018 |
| Schipp C Frontiers 2018 | Tsuchida N Arthritis Res Ther 2019 |
| Schmid JP Blood 2011 | Uzel G J Allergy Clin Immunol 2013 |
| Schreiner F JCEM 2016 | Van Eyck L Jr J Allergy Clin Immunol 2015 |
| Schubert D Nat Med 2014 | Van Leeuwen EM Journal of Clinical Immunology 2018 |
| Schwab C J Allergy Clin Immunol 2018 | Vieth S Klin Padiatr 2013 |
| Sediva H Horm Res Paediatr 2017 | Wada T Cytokine 2014 |
| Seidel MG J Allergy Clin Immunol 2015 | Wallace JG J Allergy Clin Immunol 2020 |
| Semo Oz R Pediatric Rheumatology 2019 | Wang W Pediatric Rheumatology 2019 |
| Serwas NK Inflamm Bowel Dis 2015 | Wang Y J Clin Immunol 2018 |
| Serwas NK Nat Commun 2019 | Watson LR Allergy Asthma Clin Immunol 2018 |
| Shabani M Pediatr Blood Cancer 2019 | Weinreich MA Frontiers 2017 |
| Shamriz O Eur J Pediatr 2018 | Worth AJJ Blood 2013 |
| Sharapova J Clin Immunol 2018 | Xu T Eur J Pediatr 2020 |
| Sharfe N J Allergy Clin Immunol 2014 | Yang X J Clin Immunol 2012 |
| Shields CL JAMA Ophthalmol 2016 | Yang X J Clin Immunol 2015 |
| Shiota M J Clin Immunol 2015 | Yiang MY Exp Therap Med 2016 |
| Shokri S Acta Med Iran 2016 | Zeissig S Gut 2015 |
| Sic H Front Immunol 2017 | Zerbe CS Clin Infect Dis 2016 |
| Siddiqi AE J Clin Immunol2020 | Zeynep B Eye & Contact Lens 2016 |
| Siggs OM Front Immunol 2019 | Zhang Z JEM 2019 |
| Siklar Z Exp Clin Endocrinol Diabetes 2018 | Zhao M Haematologica 2010 |
| Siobhan O J Allergy Clin Immunol 2012 | Zheng C Infamm Bowel Dis 2018 |
| Slatter MA J Allergy Clin Immunol 2016 |  |
| Snow AL JEM 2012 |  |
| Soler-Palacín P Front Immunol 2018 |  |

**Supplementary table 3. Monoclonal antibodies used for flow cytometry protocols**

| **PROTOCOL** | **FITC** | **PE** | **PC5.5** | **PC7** | **APC** | **APC-AF750** | **PB** | **KO** |
| --- | --- | --- | --- | --- | --- | --- | --- | --- |
| **SAP expression** | anti-SAP* | anti-CD16+CD56 | anti-CD8 | anti-CD19 | anti-CD3 | - | anti-CD14 | - |
| **NKG2D expression** | - | anti-NKG2D | - | anti-CD56 | anti-CD45 | anti-CD8 | anti-CD3 | - |
| **AKT phosphorylation** | - | anti-CD19 | - | - | anti-AKT (pS473) | - | - | - |
| **Senescence T cells** | anti-CCR7 | anti-CD57 | anti-CD3 | anti-CD45RA | anti-CD8 | anti-CD4 | - | - |
| **XIAP expression** | anti-XIAP* | anti-CD16+CD56 | anti-CD8 | anti-CD19 | anti-CD3 | - | anti-CD14 | - |
| **iNKT compartment** | anti-Vα24 | anti-Vβ11 | - | anti-CD56 | anti-CD3 | - | - | - |
| **Regulatory T cells** | anti-CD3 | anti-CD25 | - | - | anti-FoxP3 | - | - | anti-CD4 |
| **CTLA4 expression** | - | anti-CTLA4 | anti-CD127 | - | anti-CD3 | anti-CD25 | - | anti-CD4 |
| **LRBA expression** | anti-CD8 | - | anti-CD20 | - | anti-LRBA* | anti-CD4 | anti-CD3 | anti-CD45 |
| **Follicular T cells** | anti-CXCR5 | anti-CXCR3 | anti-CD3 | anti-PD1 | - | anti-CD45RA | anti-CCR6 | anti-CD4 |
| **STAT3 phosphorylation** | - | anti-STAT3 (pY705) | - | - | anti-CD3 | - | - | - |
| **STAT1 phosphorylation** | anti-CD14 | - | anti-STAT1 (pY701) | - | - | - | - | - |

* Unconjugated + secondary conjugated anti-IgG

**Supplementary table 4. Hematopoietic stem cell transplantation in ALPS-like patients**

| **ALPS-like disorder** | **Positive effect** | **Negative effect** | **Total HSCT** | **% HSCT*** | **References** |
| --- | --- | --- | --- | --- | --- |
| ADA2 deficiency | 7 | 0 | 7 | 22.6 | (1–5) |
| CARD11 GOF | 1 | 0 | 1 | 6.3 | (6) |
| CTLA4 haploinsufficiency | 19 | 10 | 29 | 18.8 | (7–10) |
| CD25 deficiency | 1 | 0 | 1 | 25 | (11) |
| CD122 deficiency | 1 | 1 | 2 | 50 | (12) |
| LRBA deficiency | 25 | 4 | 29 | 19.2 | (13–22) |
| PIK3CD GOF + PIK3R1 LOF | 4 | 0 | 4 | 12.1 | (23–26) |
| RASGRP1 deficiency | 2 | 0 | 2 | 40 | (27) |
| SH2D1A deficiency | 3 | 0 | 3 | 12 | (28,29) |
| STAT1 GOF | 1 | 0 | 1 | 7.7 | (30) |
| STAT3 GOF | 1 | 1 | 2 | 4.5 | (31) |
| STK4 deficiency | 1 | 0 | 1 | 8.3 | (32) |
| TET2 deficiency | 1 | 2 | 3 | 100 | (33) |
| TNFRSF9 deficiency | 1 | 0 | 1 | 33.3 | (34) |
| TPP2 deficiency | 1 | 0 | 1 | 50 | (35) |
| XIAP deficiency | 13 | 8 | 21 | 32.8 | (36–44) |
| **Total patients** | **82** | **26** | **108** |  |  |

*percentage of transplanted patients in respect to the total number of considered ALPS-like patients in each disorder (see supplementary table 1)
