# Supplementary figures and images for: Primary Immune Regulatory Disorders With an Autoimmune Lymphoproliferative Syndrome-Like Phenotype: Immunologic Evaluation, Early Diagnosis and Management
Source: Front Immunol. 2021 Aug 10;12:671755. doi: 10.3389/fimmu.2021.671755 (PMC8382720; doi:10.3389/fimmu.2021.671755)

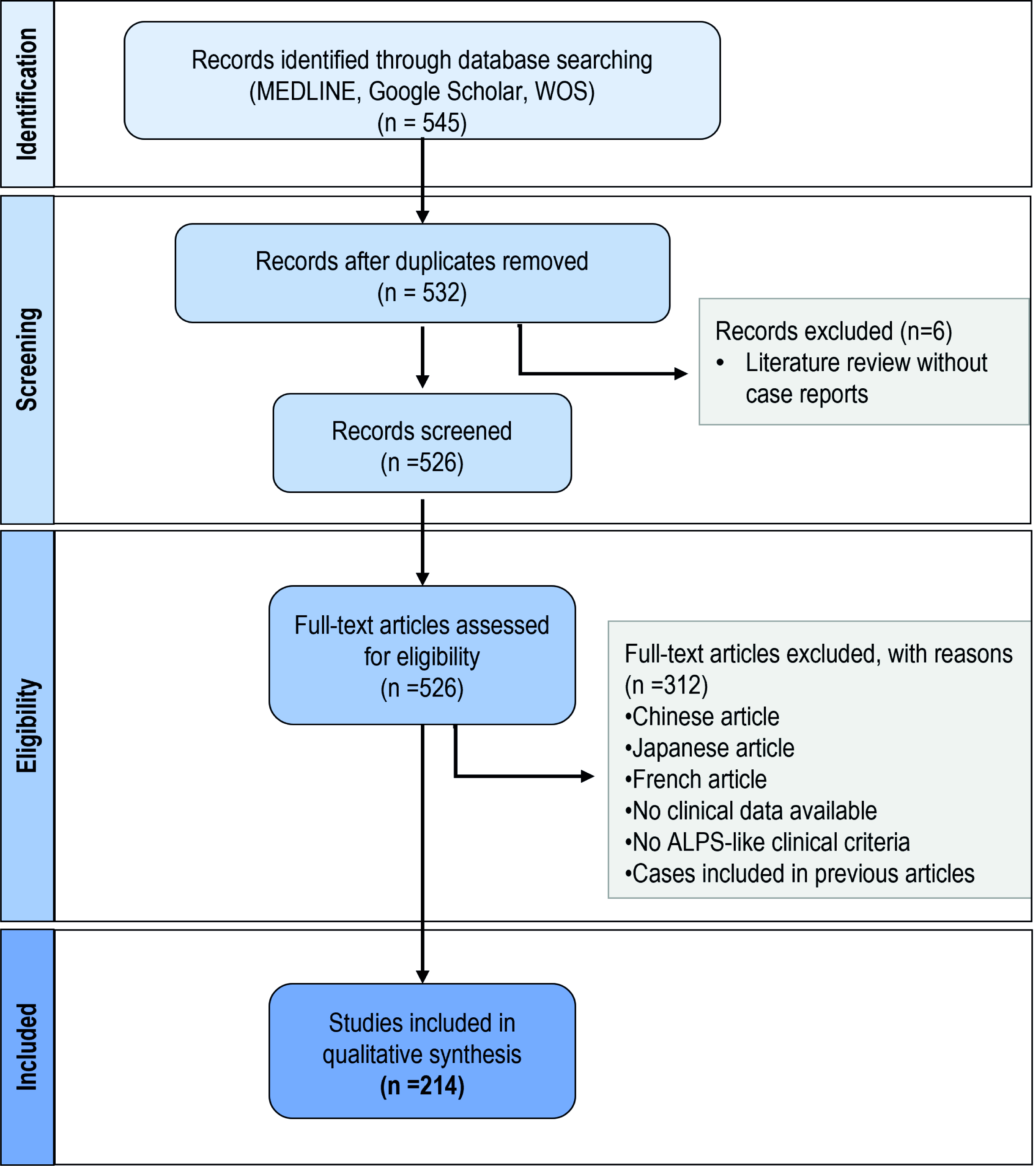

Supplement: Supplementary file 2 [file Image_1.jpg]

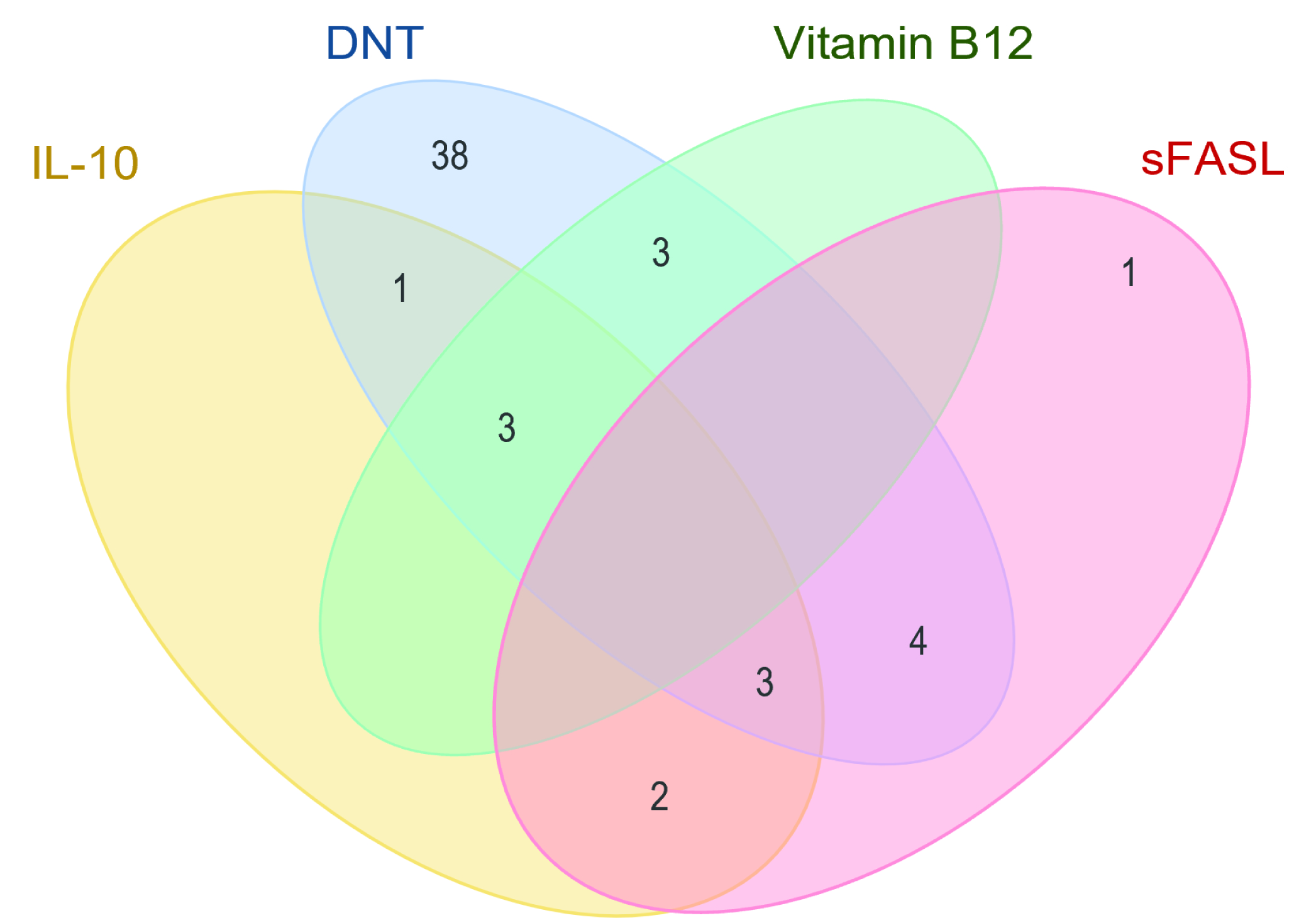

Supplement: Supplementary file 3 [file Image_2.jpg]
